# Supplementary material for: Structure-based rational design of an enhanced fluorogen-activating protein for fluorogens based on GFP chromophore
Source: Commun Biol. 2022 Jul 15;5:706. doi: 10.1038/s42003-022-03662-9 (PMC9287381; doi:10.1038/s42003-022-03662-9)
Supplement: Supplementary file 2 — Supplementary Information [file 42003_2022_3662_MOESM2_ESM.pdf]

SUPPLEMENTARY MATERIALS

Structure-based rational design of an enhanced fluorogen-activating protein for fluorogens based on GFP chromophore

Marina V. Goncharuk[1,5], Nadezhda S. Baleeva[1,5], Dmitry E. Nolde[1,3], Alexey S. Gavrikov[1], Alexey V. Mishin[2], Alexander S. Mishin[1], Andrey Y. Sosorev[1], Alexander S Arseniev[1], Sergey A. Goncharuk[1,2], Valentin I. Borshchevskiy[2], Roman G Efremov[1,2,3], Konstantin S. Mineev[1,2,6]*, Mikhail S. Baranov[1,4,6]*

*corresponding authors: mineev@nmr.ru, baranovmikes@gmail.com

[1]Shemyakin-Ovchinnikov Institute of Bioorganic Chemistry RAS, Moscow 117997, Russia

[2]Moscow Institute of Physics and Technology, Dolgoprudny 141701, Russia

[3]National Research University Higher School of Economics, Moscow 101000, Russia

[4]Pirogov Russian National Research Medical University, Moscow 117997, Russia

[5]These authors contributed equally: Marina V. Goncharuk and Nadezhda S. Baleeva

[6]These authors jointly supervised the work: Konstantin S. Mineev and Mikhail S. Baranov

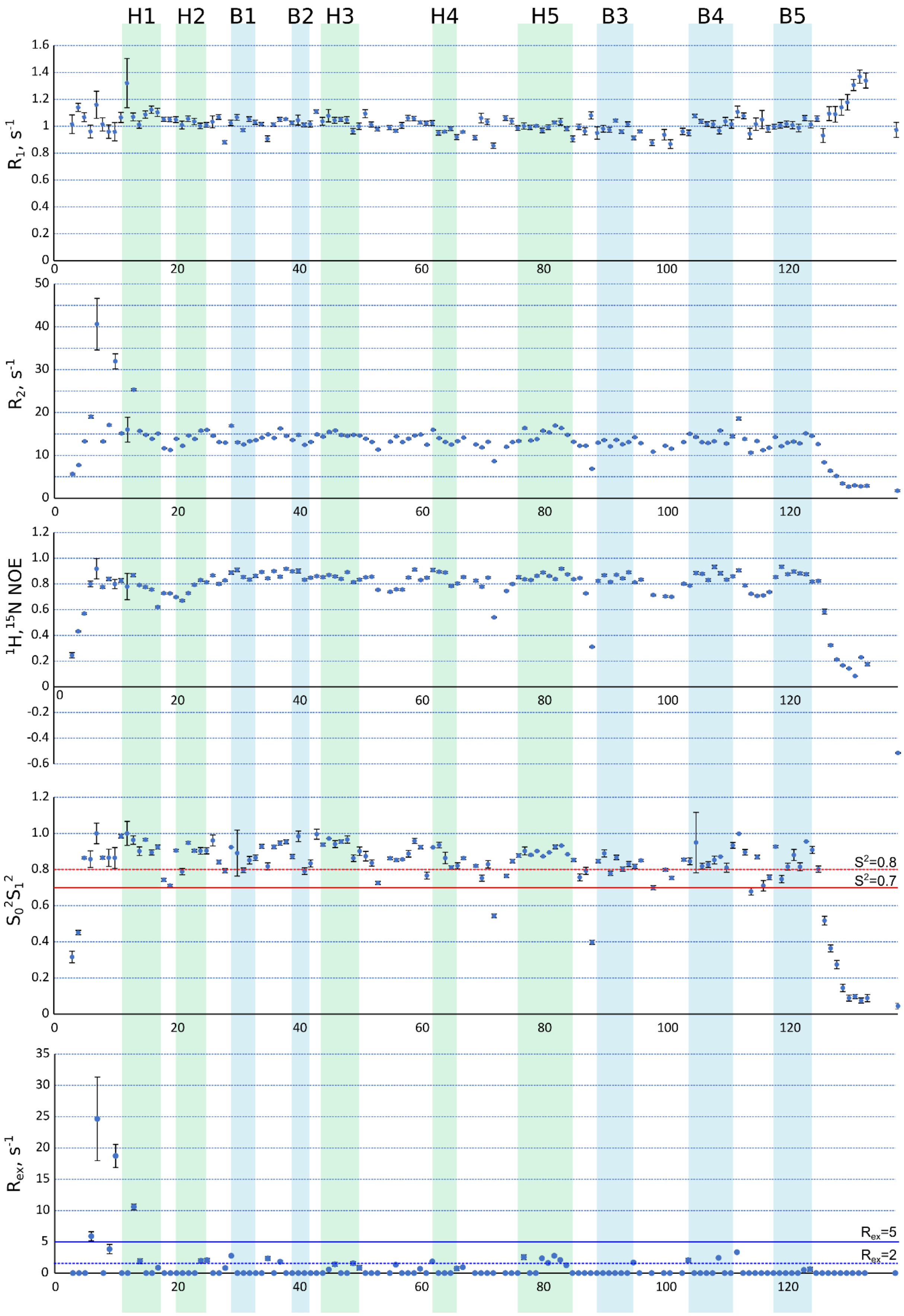
H1
H2
B1
B2
H3
H4
H5
B3
B4
B5
R1, s-1
R2, s-1
1H,15N NOE
S02S12
S2=0.8
S2=0.7
Rex, s-1
Rex=5
Rex=2

Supplementary Figure 1. ^{15}N NMR relaxation parameters of FAST/**N871b**. Rates of longitudinal (R_1) and transverse (R_2) relaxation, ^1H,^{15}N heteronuclear steady-state NOE, generalized order parameters of NH groups ($S_0^2S_1^2$) and contribution of μs-ms motions to the transverse relaxation (R_{ex}) are plotted for the individual residues of FAST in complex with **N871b**. All parameters were measured at 25°C at 700 MHz. S_0^2 and S_1^2 are the order parameters of subnanosecond and nanosecond motions, respectively. The order parameter and R_{ex} thresholds used to color the structure in Figure 1 are shown as dashed and solid blue and red lines. Error bars in correspond to the errors of approxiamtion, determined by the Monte-Carlo analysis.

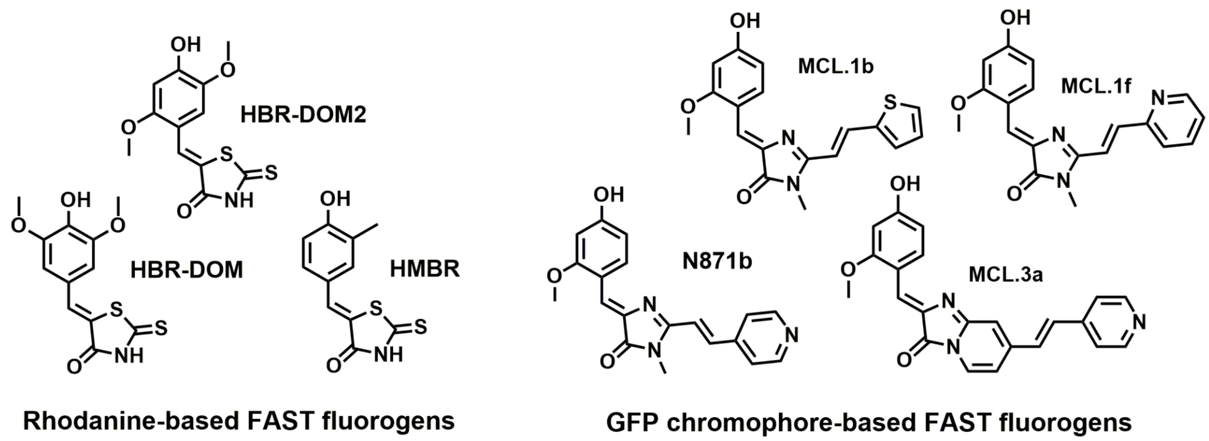

Supplementary Figure 2. Chemical structures of rhodanine-based conventional FAST fluorogens, and GFP chromofore-based fluorogens, studied here.

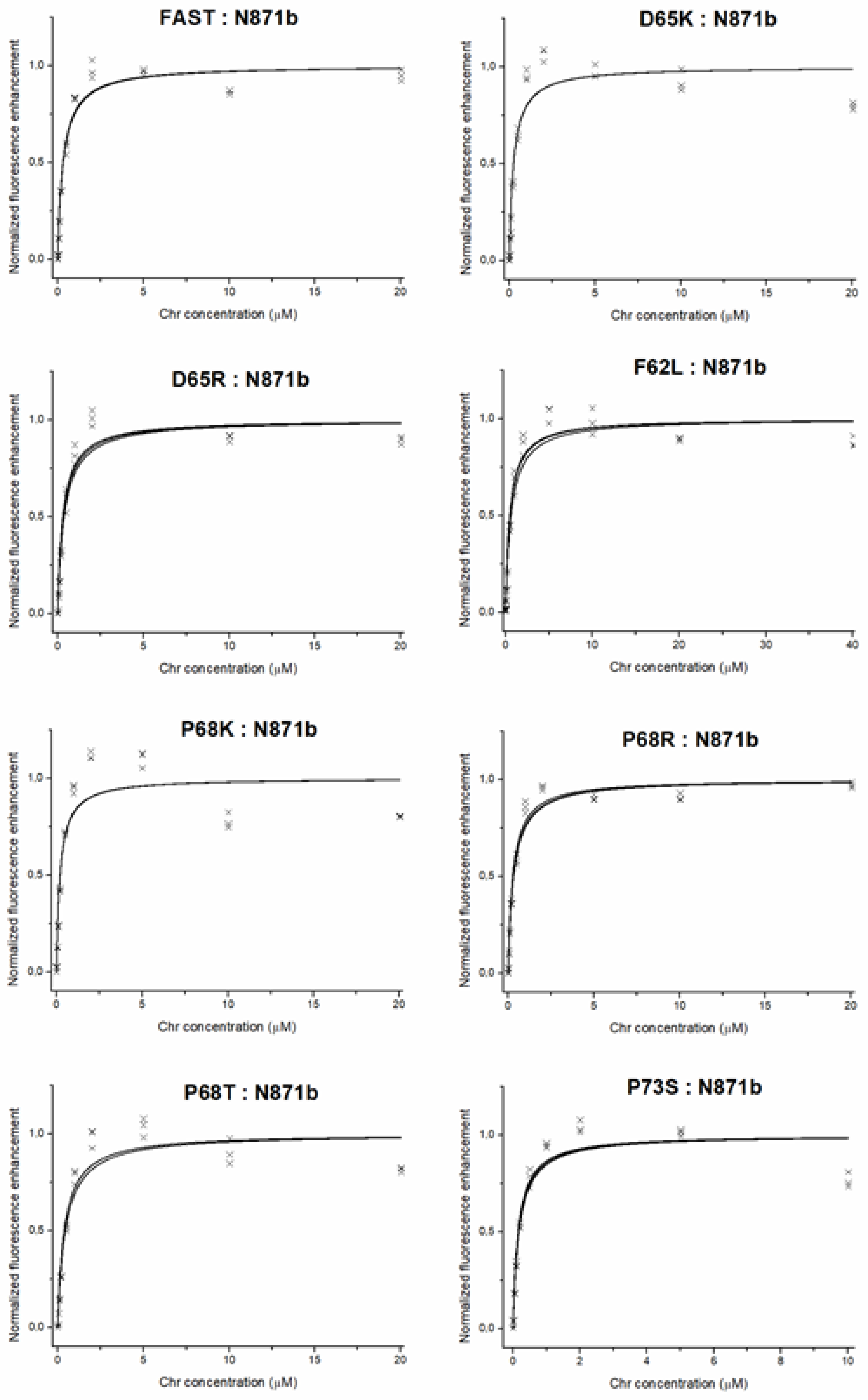

Supplementary Figure 3. Titration curves observed for **N871b** complexes with FAST mutants measured in PBS buffer.

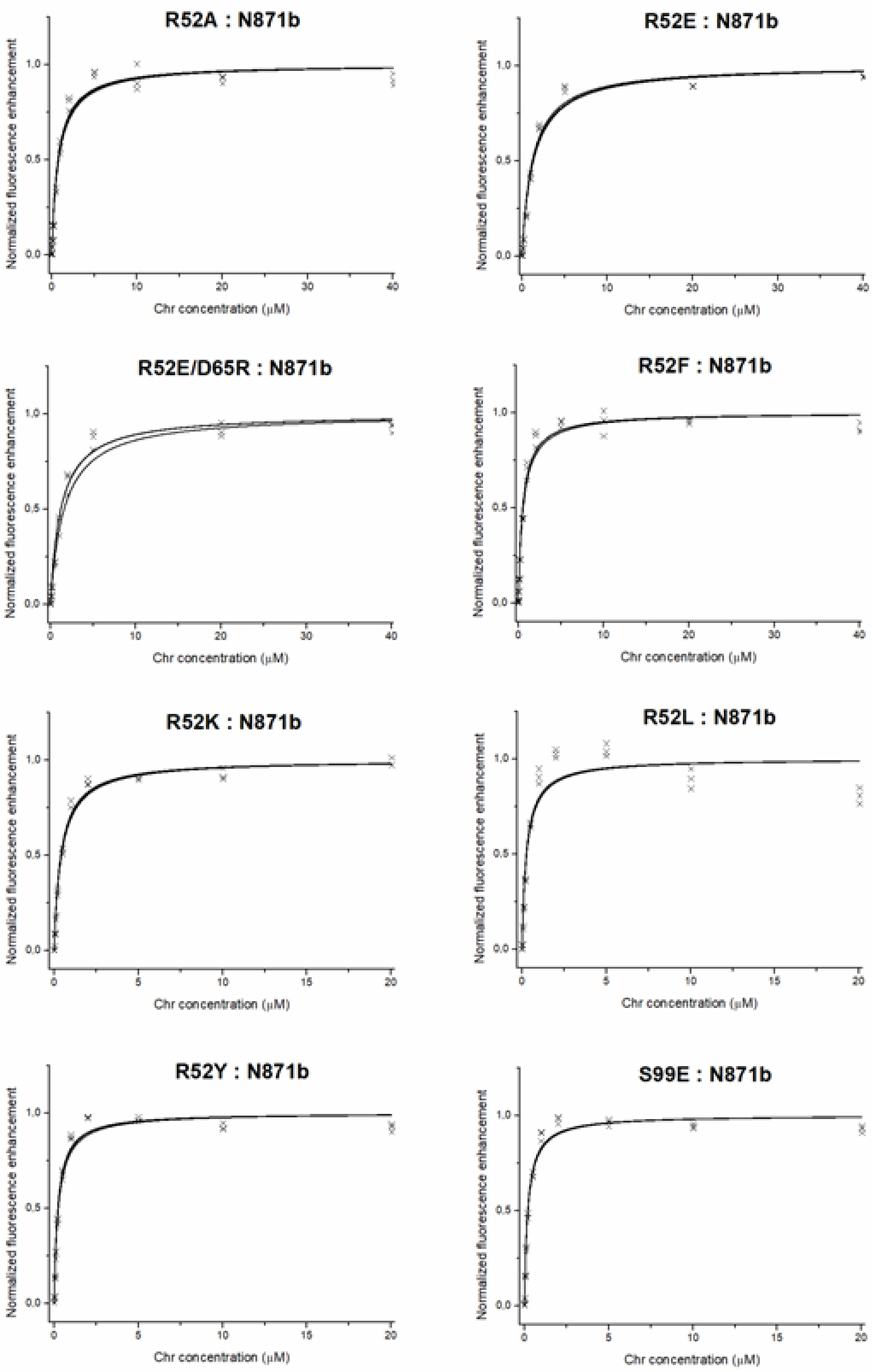

Supplementary Figure 4. Titration curves observed for **N871b** complexes with FAST mutants measured in PBS buffer.

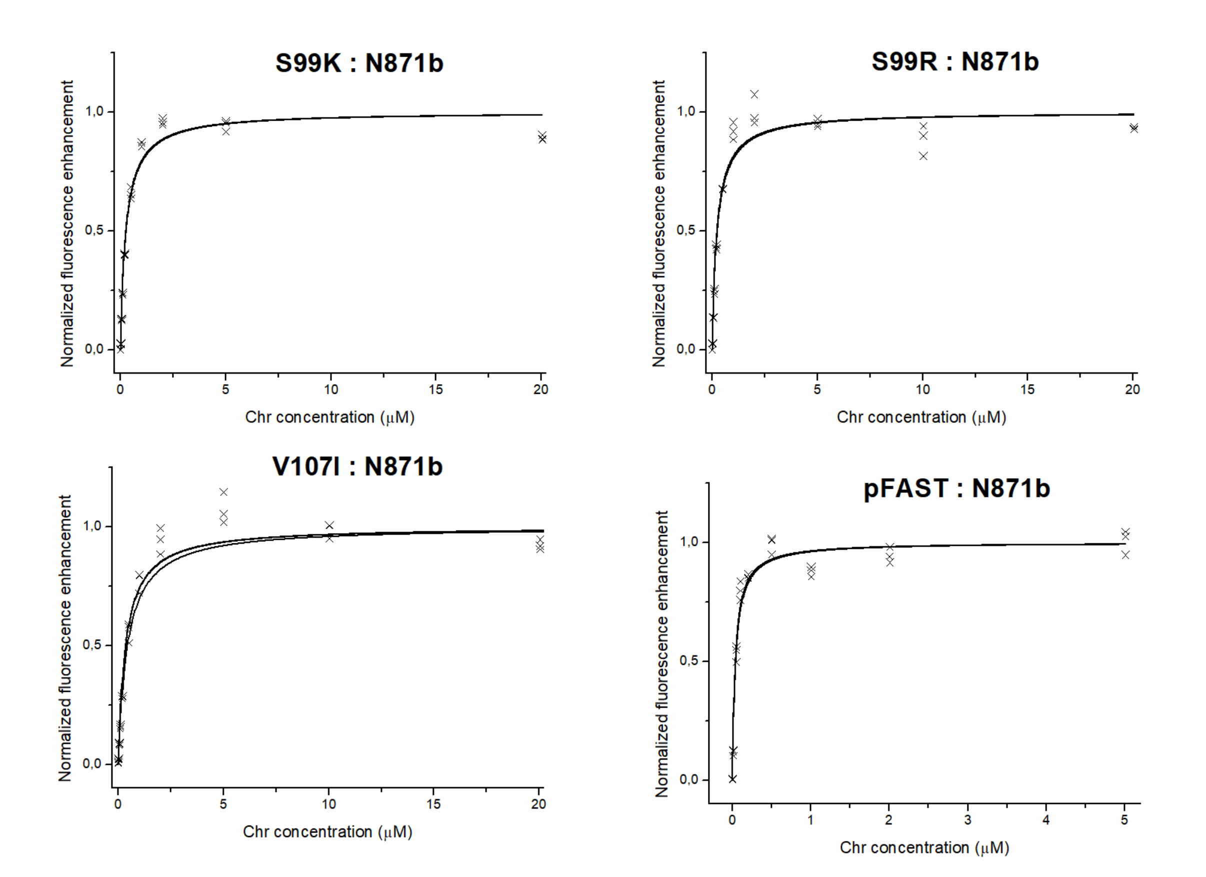

Supplementary Figure 5. Titration curves observed for **N871b** complexes with FAST mutants measured in PBS buffer.

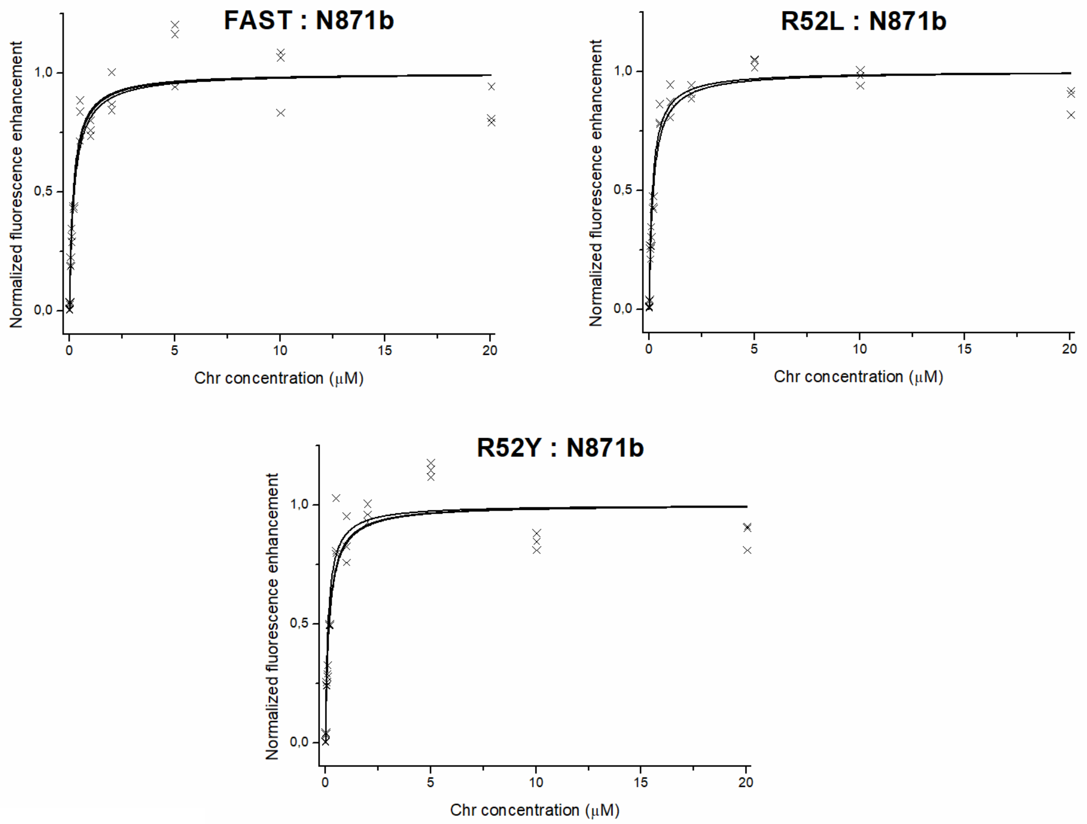

Supplementary Figure 6. Titration curves observed for **N871b** complexes with FAST mutants in the NMR buffer (pH 7.0, 20 mM NaPi, 20 mM NaCl).

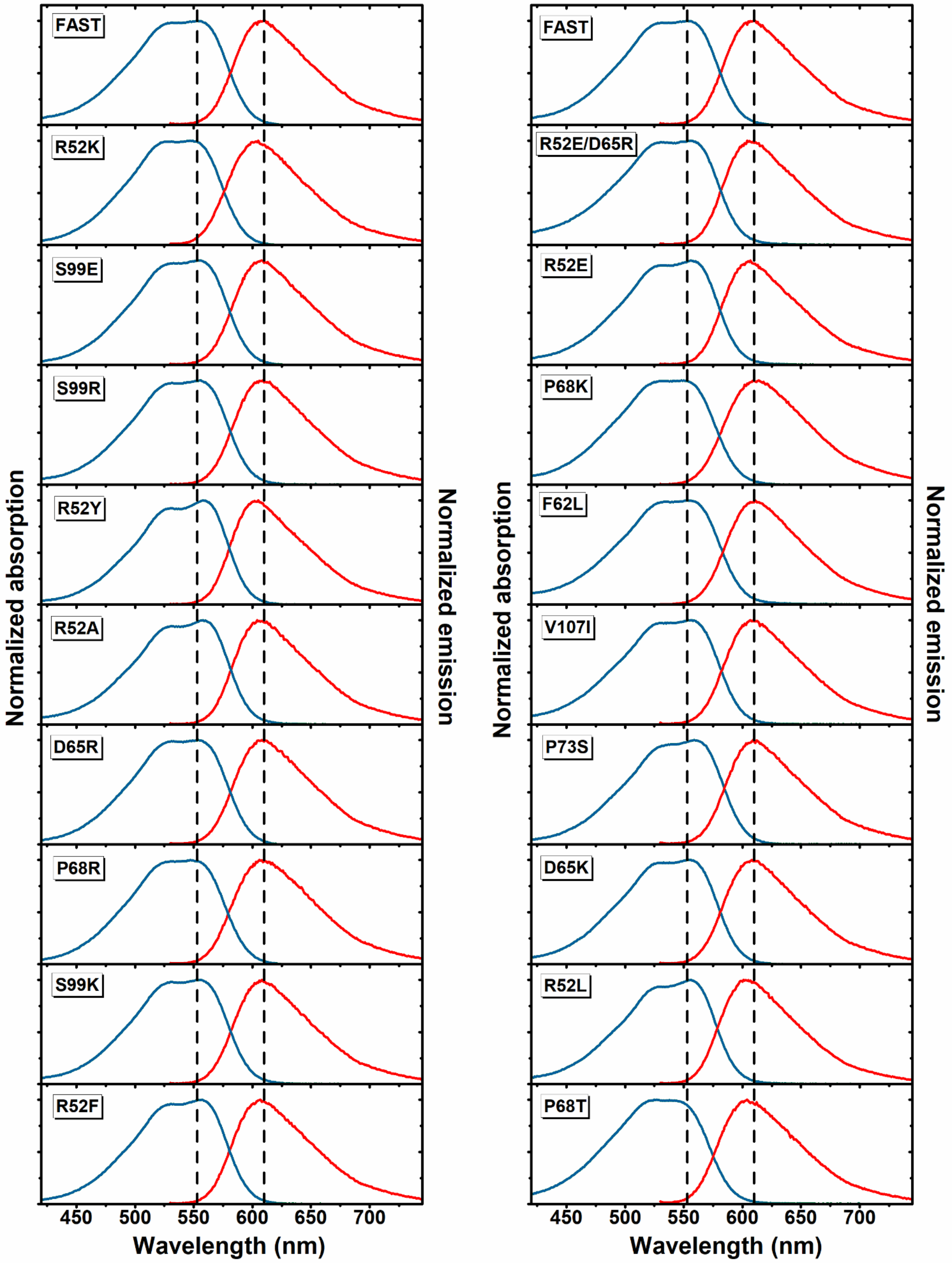

Supplementary Figure 7. Fluorescence (red) and absorption (blue) spectra of **N871b** complexes with FAST mutants measured in PBS buffer.

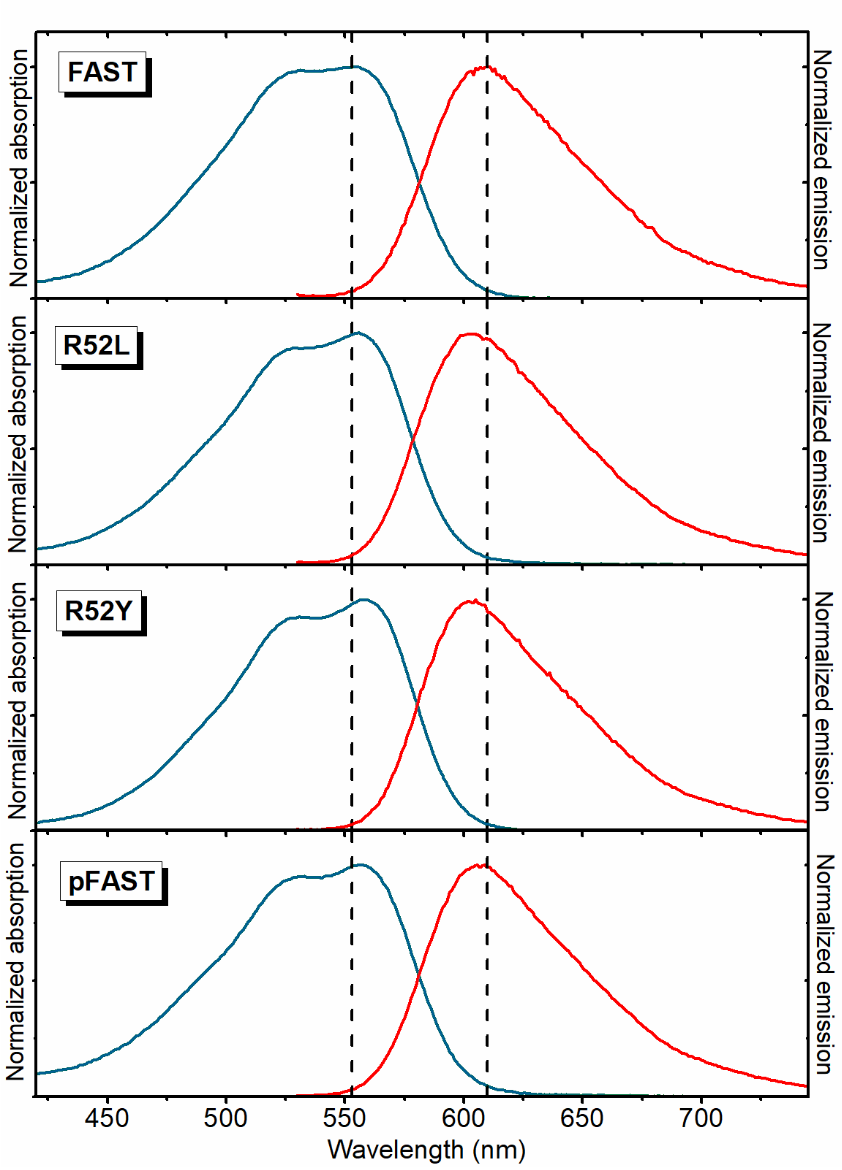

Supplementary Figure 8. Fluorescence (red) and absorption (blue) spectra of **N871b** complexes with FAST mutants measured in PBS buffer.

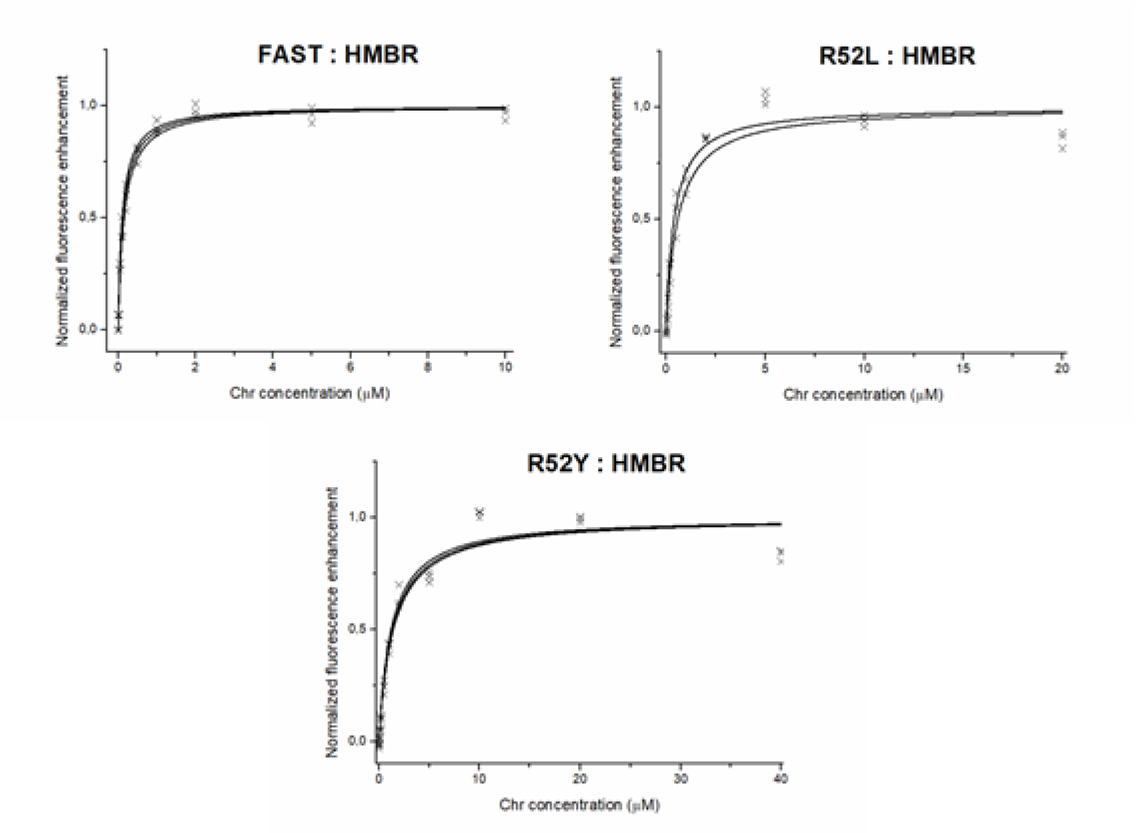

Supplementary Figure 9. Titration curves observed for **HMBR** complexes with FAST mutants measured in PBS buffer.

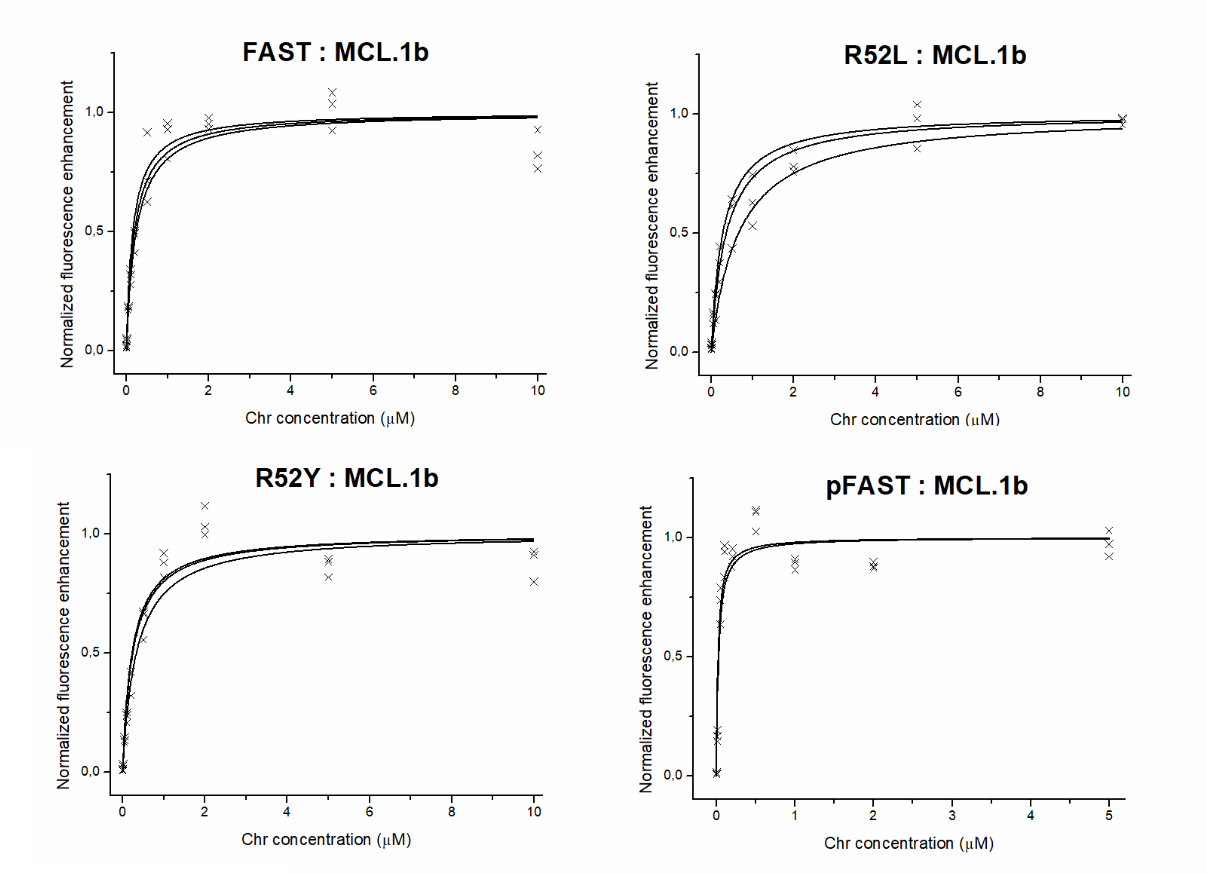

Supplementary Figure 10. Titration curves observed for **MCL.1b** complexes with FAST mutants measured in PBS buffer.

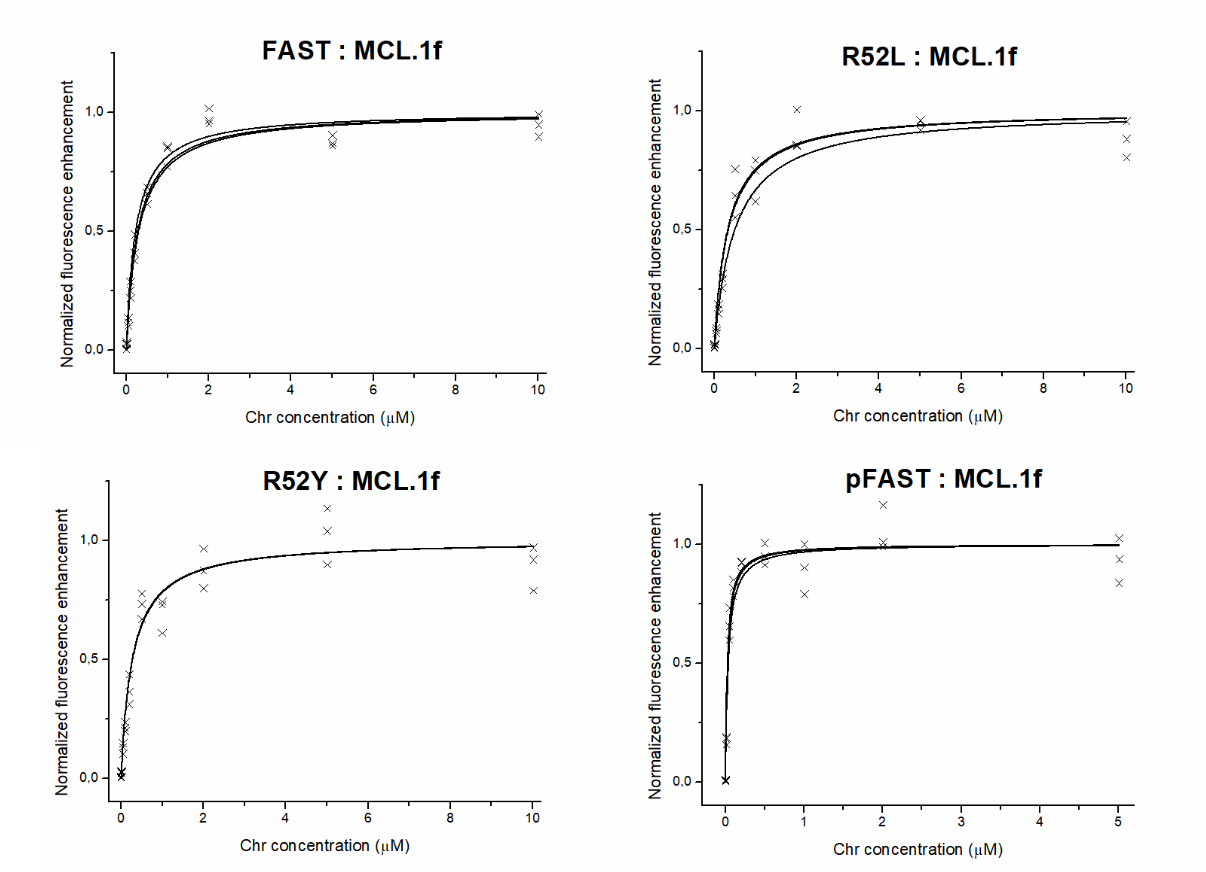

Supplementary Figure 11. Titration curves observed for **MCL.1f** complexes with FAST mutants measured in PBS buffer.

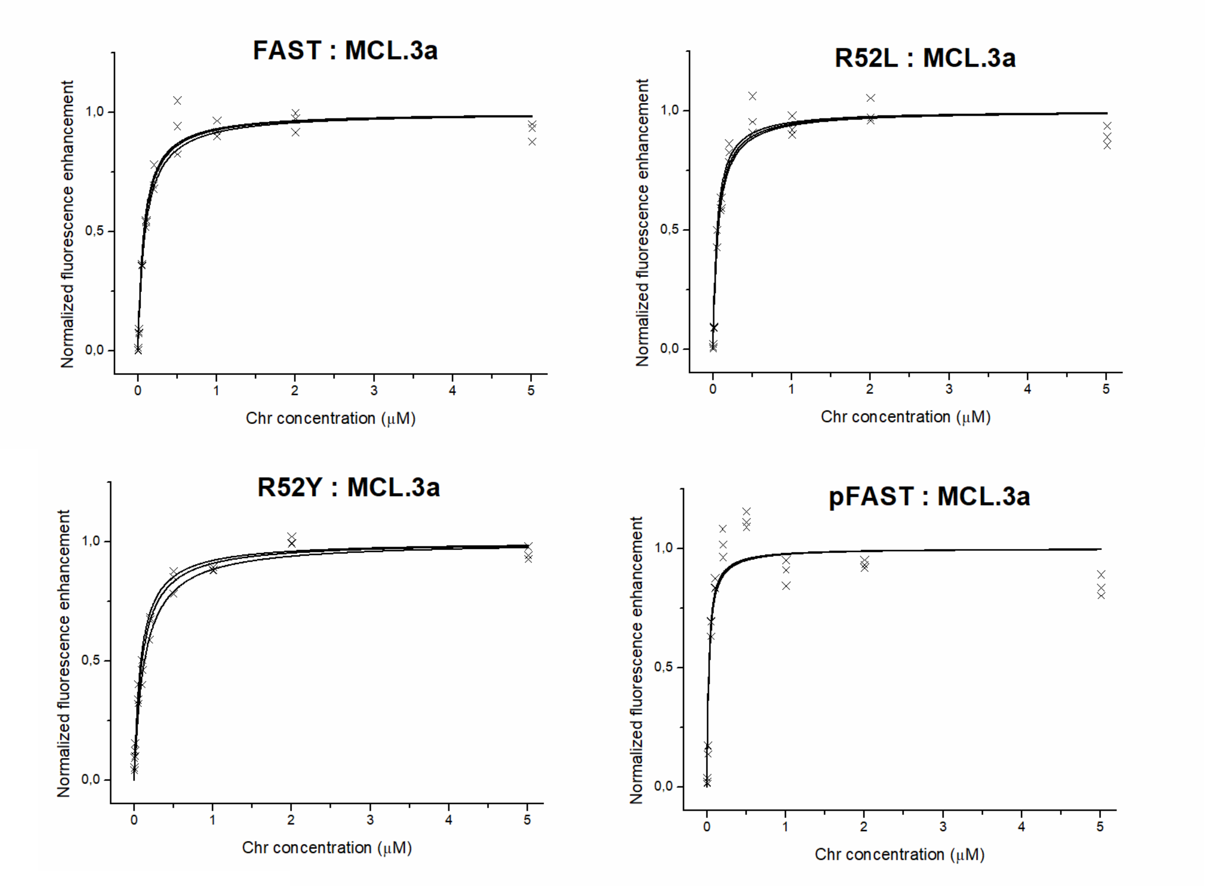

Supplementary Figure 12. Titration curves observed for **MCL.3a** complexes with FAST mutants measured in PBS buffer.

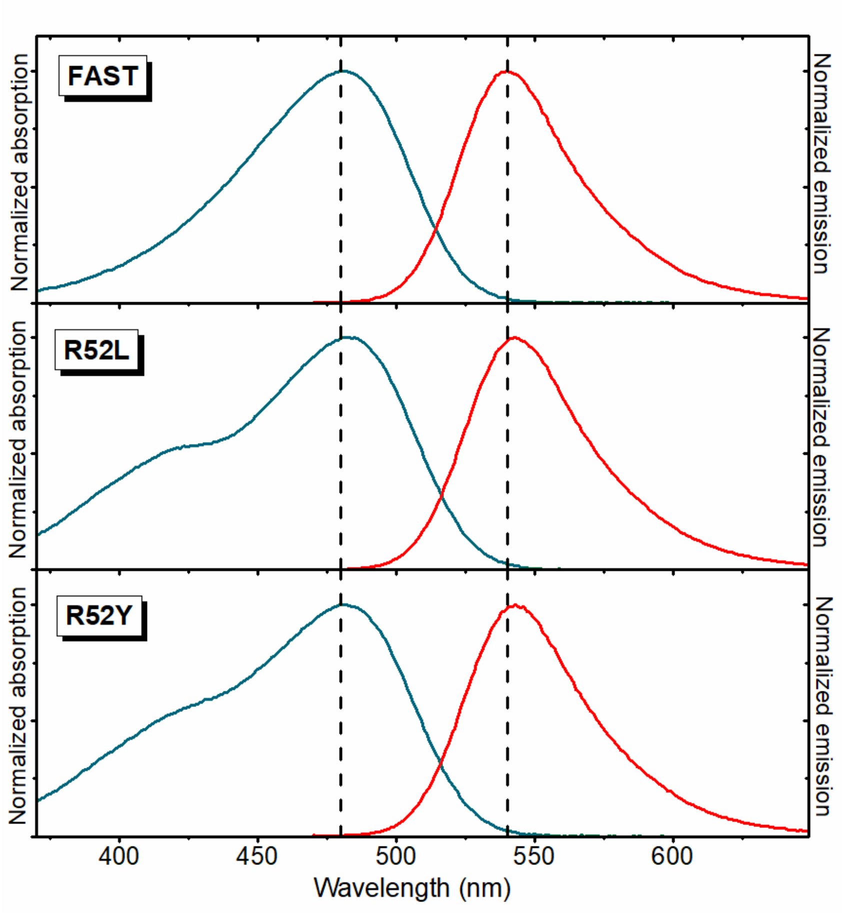

Supplementary Figure 13. Fluorescence (red) and absorption (blue) spectra of **HMBR** complexes with FAST mutants measured in PBS buffer.

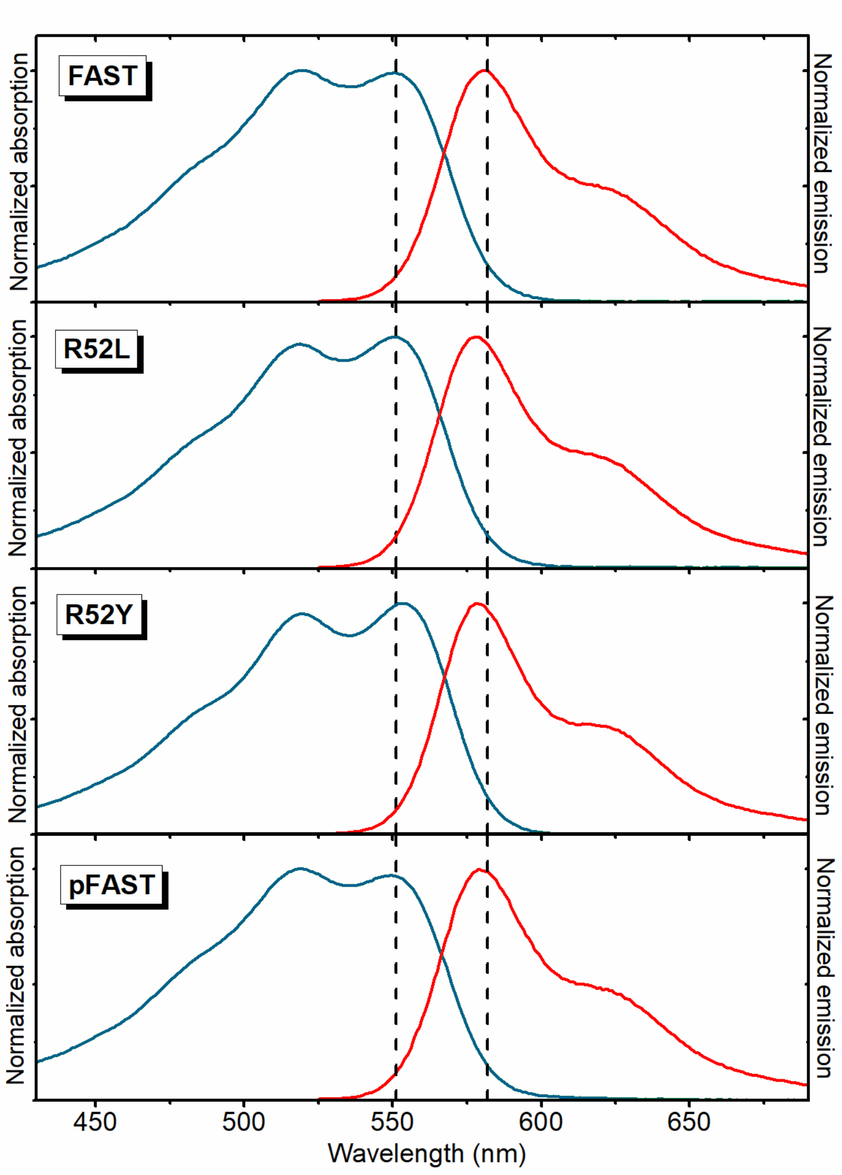

Supplementary Figure 14. Fluorescence (red) and absorption (blue) spectra of **MCL.1b** complexes with FAST mutants measured in PBS buffer.

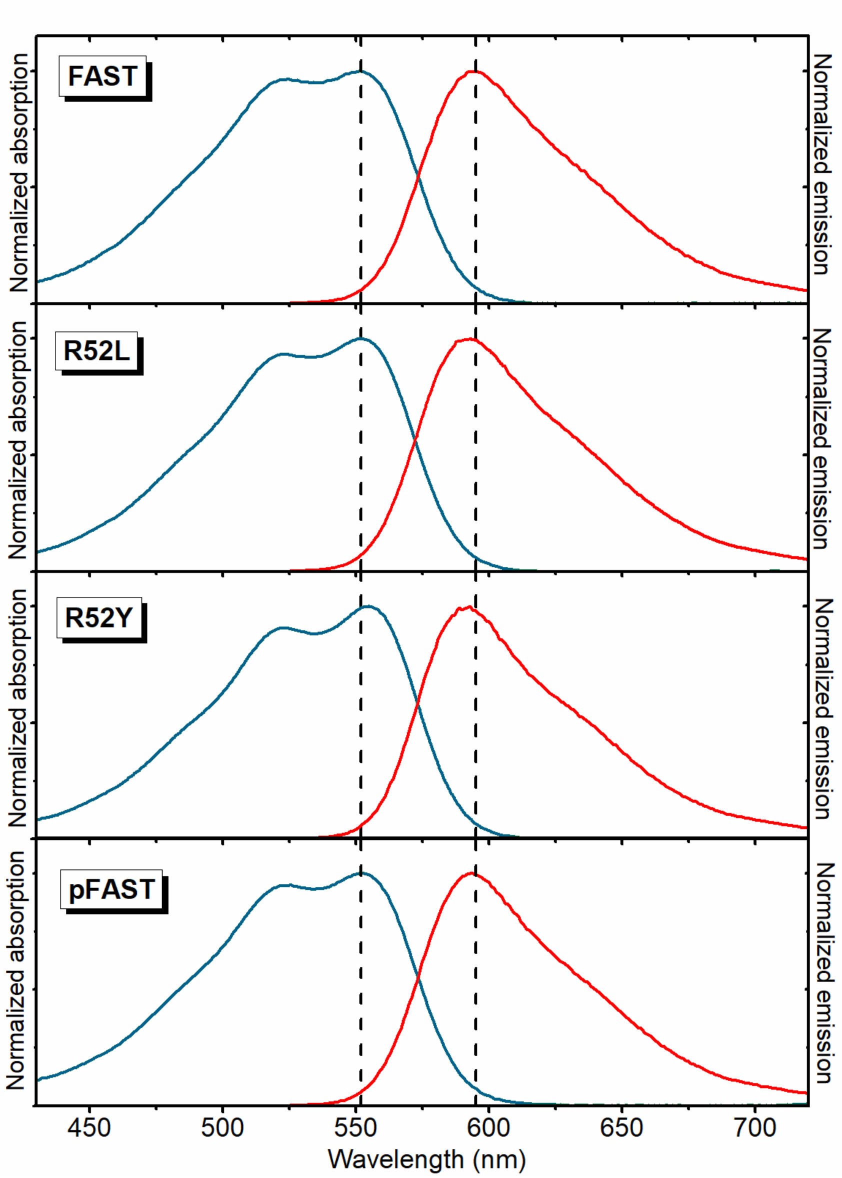

Supplementary Figure 15. Fluorescence (red) and absorption (blue) spectra of **MCL.1f** complexes with FAST mutants measured in PBS buffer.

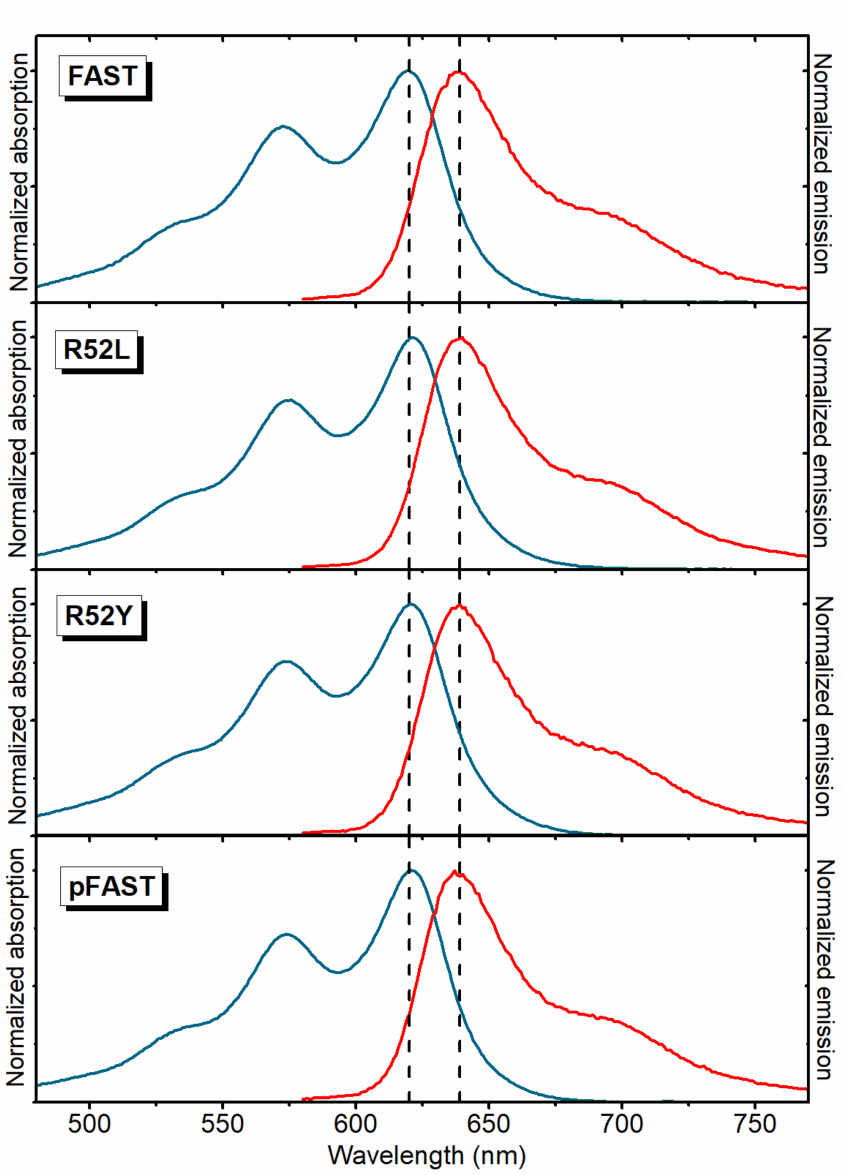

Supplementary Figure 16. Fluorescence (red) and absorption (blue) spectra of **MCL.3a** complexes with FAST mutants measured in PBS buffer.

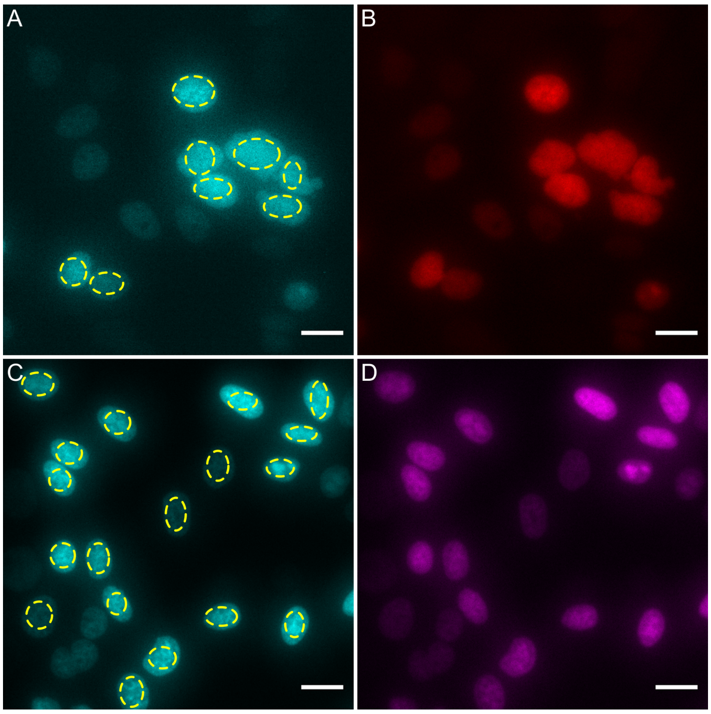

Supplementary Figure 17. Representative images of HeLa Kyoto nuclei used to evaluate relative brightness of FAST with different chromophores. HeLa Kyoto cells transiently transfected with H2B-TagBFP-mcFAST-L construct with addition of **MCL.1f** (A, B), and **MCL.3a** (C, D) chromophores imaged in Blue channel (A, C), mCherry channel (B), and CY5 channel (D). Scale bars are 20 μm. Yellow dashed circles represent selected ROIs for relative brightness evaluation.

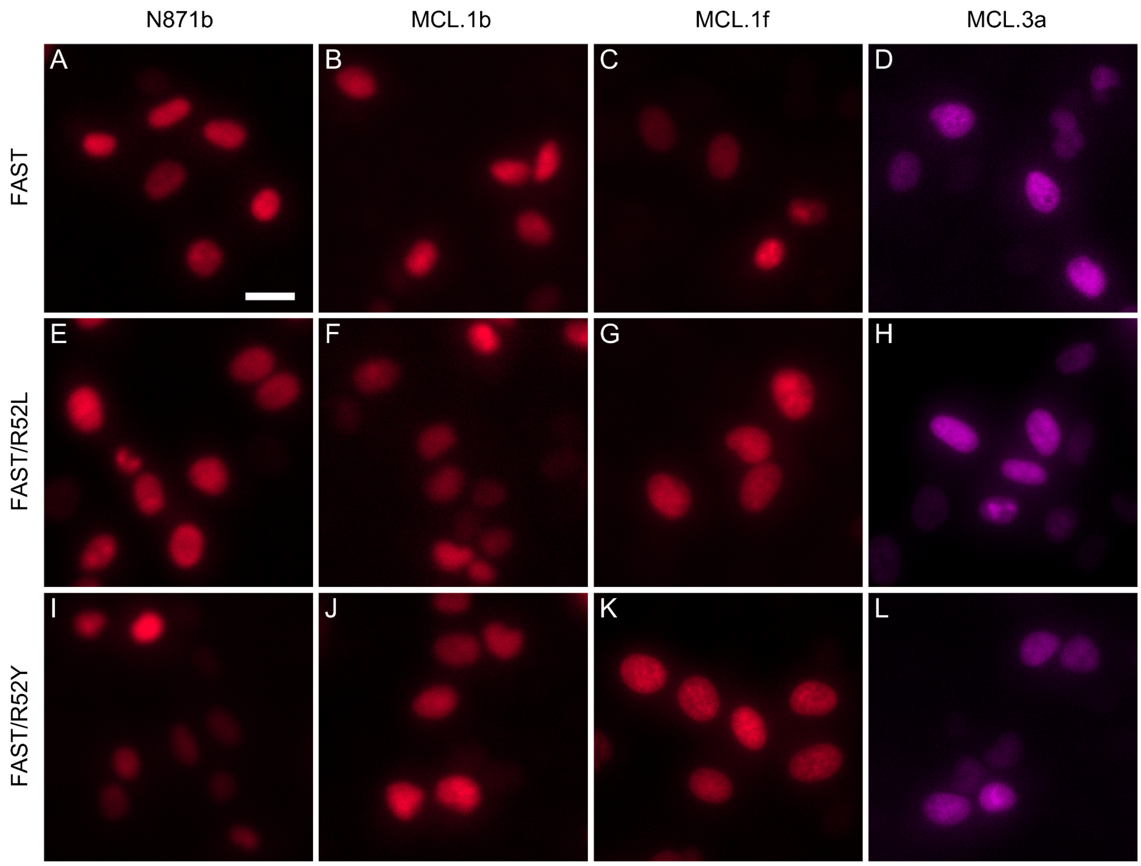

Supplementary Figure 18. Representative images of HeLa Kyoto cells transiently transfected with H2B-TagBFP-FAST (upper row), H2B-TagBFP-mcFAST-L (middle row), and H2B-TagBFP-mcFAST-Y (lower row) used to evaluate relative brightness of FAST mutant variants with different chromophores in mCherry channel (N871b, MCL.1b, MCL.1f) and CY5 channel (MCL.3a); Concentration was 20 µM for each chromophore; Scale bar is 20 µm.

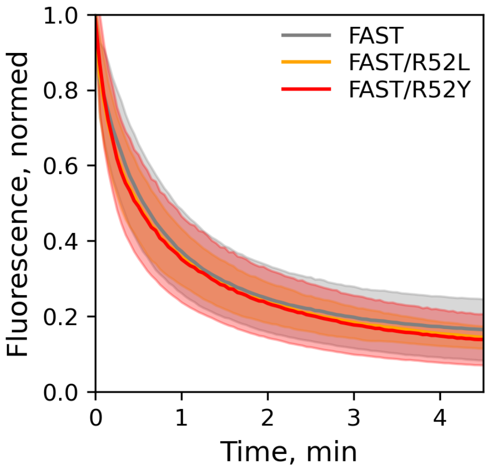

Supplementary Figure 19. Comparison of the photobleaching curves of FAST variants in the presence of 20 µM of N871b. Solid lines represent mean value, shaded - standard deviation, n = 27 nuclei for each mutant variant.

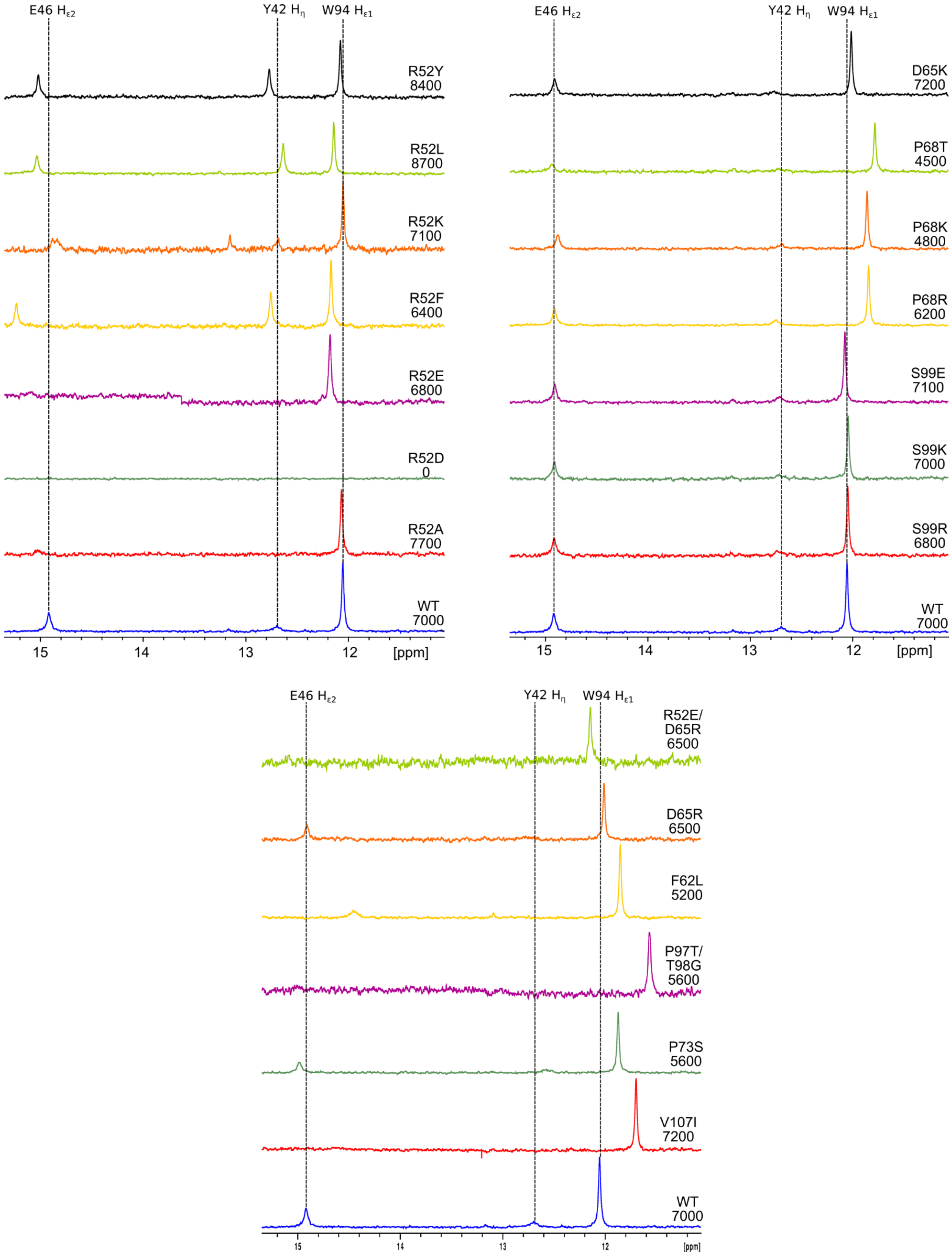

Supplementary Figure 20. The overlay of ^{1}H NMR spectra of FAST/**N871b** complexes, recorded for the wild-type protein and its mutants. Spectra were obtained at 25°C in PBS buffer, molecular brightness of the complex is indicated.

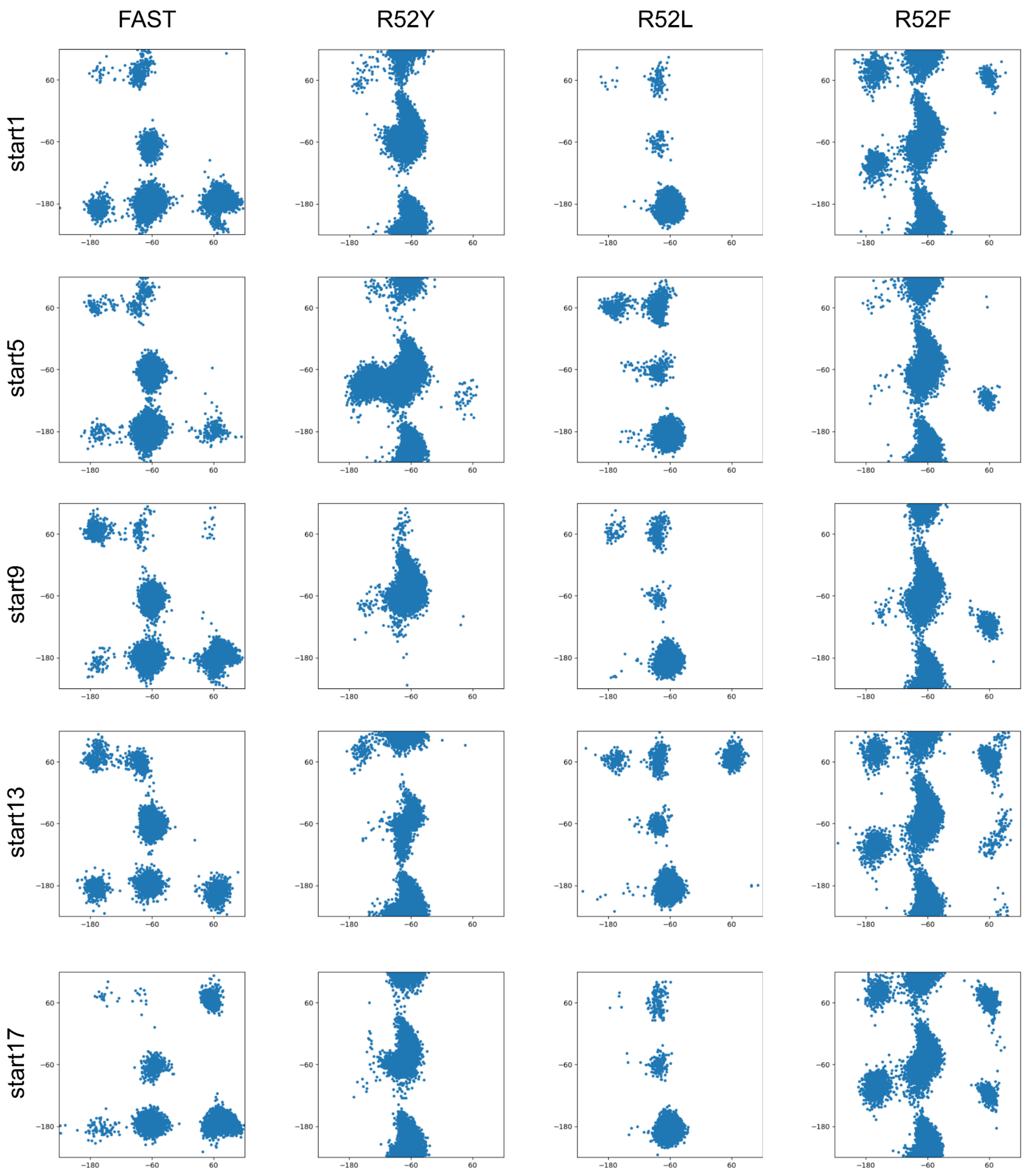

Supplementary Figure 21. χ_1/χ_2 distributions obtained for the sidechain of #52 residue in the course of 5 independent MD simulations, started from various NMR models in PDB ID 7AVA (models 1, 5, 9, 13 and 17).

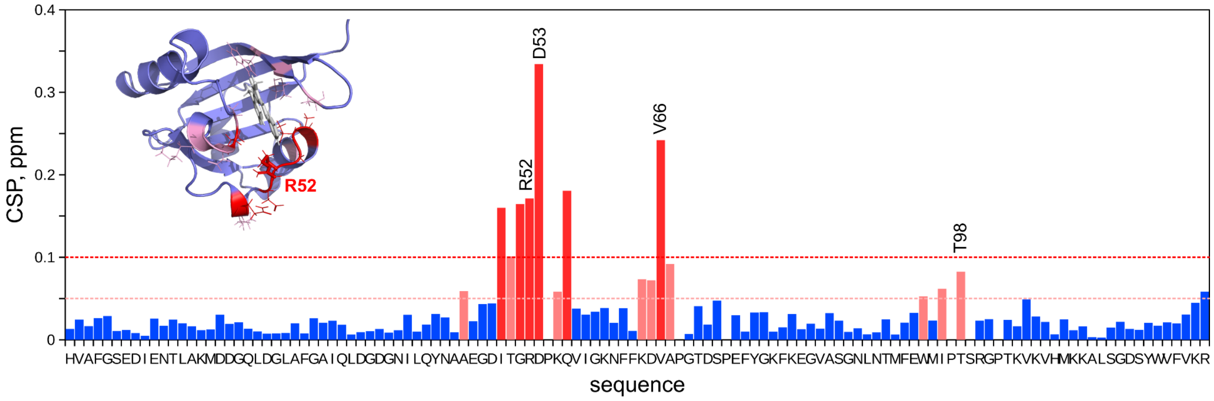

Supplementary Figure 22. NMR chemical shift perturbations (CSP) of amide resonances, induced by the R52Y mutation in FAST. Residues with CSPs exceeding 0.1 ppm are colored red, and residues with CSPs exceeding 0.05 ppm are colored pink. Structure of FAST/N871b complex, colored in the similar manner is shown for clarity, position of R52 sidechain is indicated. CSPs were calculated using the relationship: $CSP = \sqrt{\Delta\delta_H^2 + \frac{\Delta\delta_N^2}{100}}$, where $\Delta\delta_H$ and $\Delta\delta_N$ are the chemical shift changes of protons and nitrogens, respectively, while 100 is introduced to take into account the difference in gyromagnetic ratios of these two nuclei.

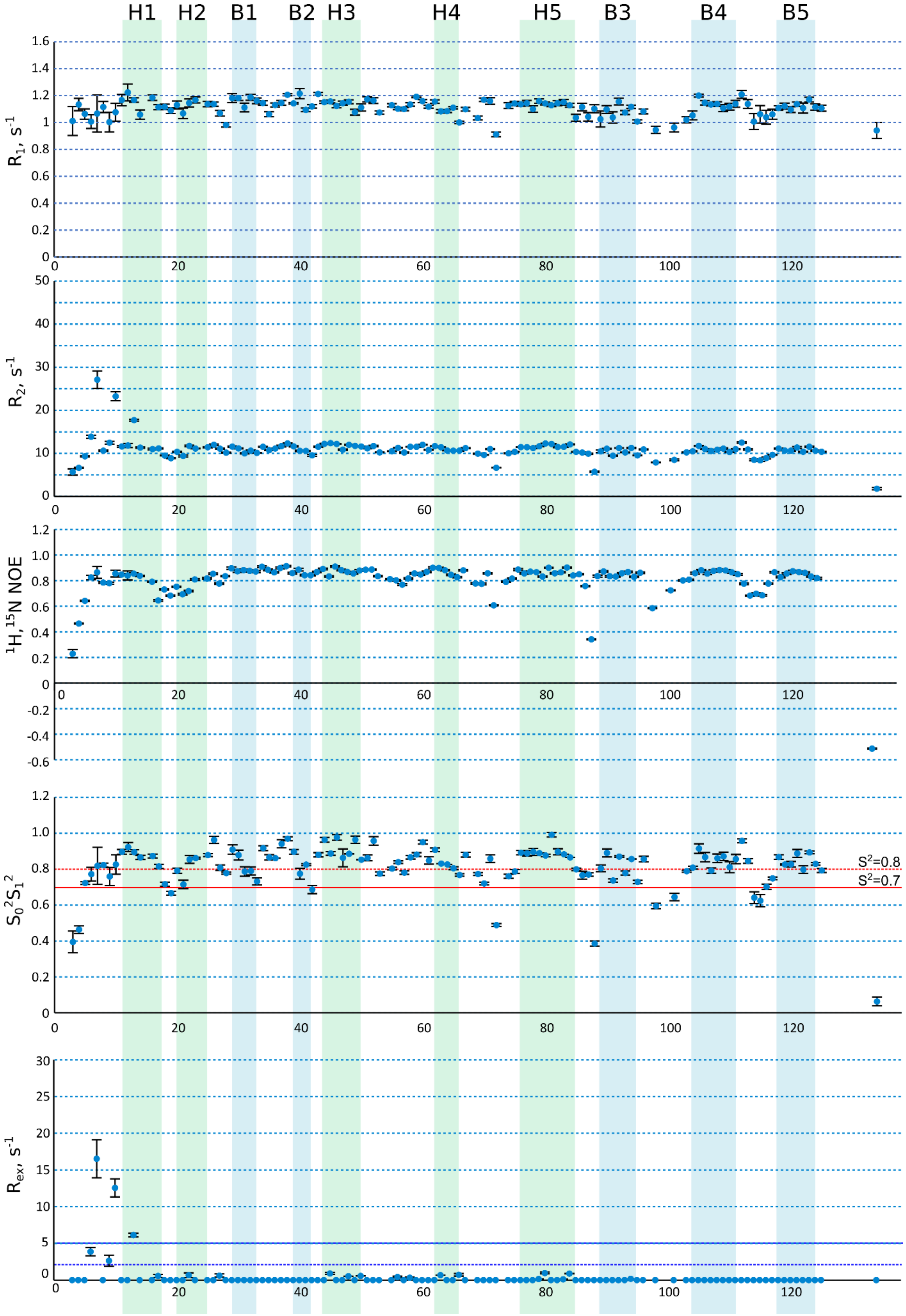

H1
H2
B1
B2
H3
H4
H5
B3
B4
B5
R1, s-1
R2, s-1
1H,15N NOE
S0 2S1 2
S2=0.8
S2=0.7
Rex, s-1

Supplementary Figure 23. ^{15}N NMR relaxation parameters of mcFAST-Y/**N871b**. Rates of longitudinal (R_1) and transverse (R_2) relaxation, ^{1}H,^{15}N heteronuclear steady-state NOE, generalized order parameters of NH groups ($S_0^2S_1^2$) and contribution of μs-ms motions to the transverse relaxation (R_{ex}) are plotted for the individual residues of FAST in complex with **N871b**. All parameters were measured at 25 °C at 700 MHz. S_0^2 and S_1^2 are the order parameters of subnanosecond and nanosecond motions, respectively. The order parameter and R_{ex} thresholds same as used to color the structure in Figure 1 are shown as dashed and solid blue and red lines. Error bars in correspond to the errors of approxiamtion, determined by the Monte-Carlo analysis.

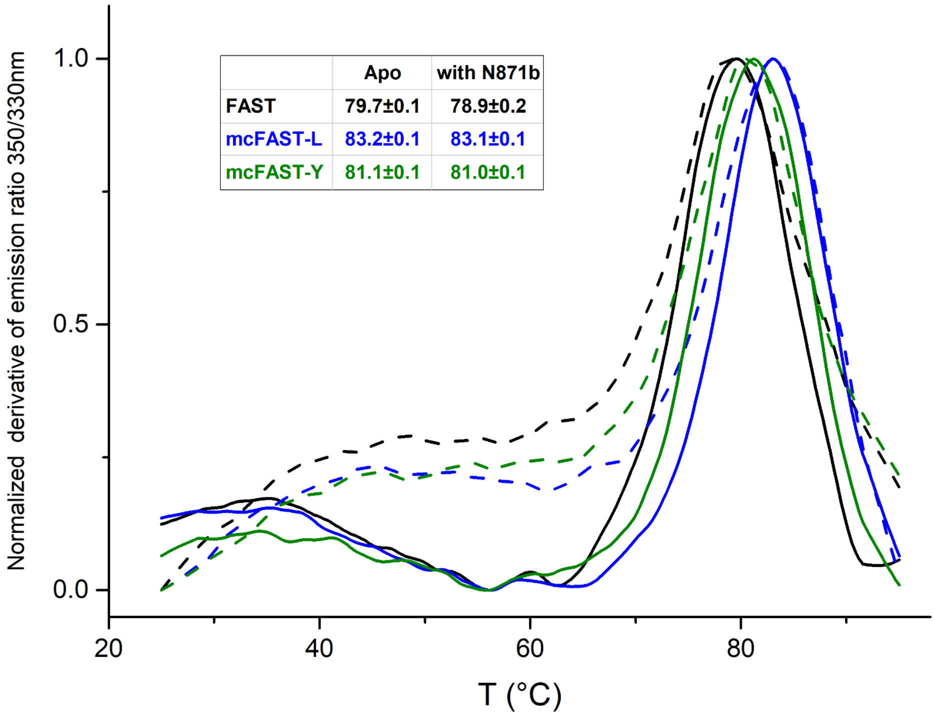

Supplementary Figure 24. Representative curves for the normalized derivative of fluorescence emission ratio at 350/330 nm as a function of temperature. Curves for FAST shown in black, mcFAST-L in blue, mcFAST-Y in green, apo forms shown as solid lines, **N871b** bound forms - as dashed lines. The table shows Tm (°C) values for FAST, mcFAST-L and mcFAST-Y both in apo and N871b bound states. Data represent the mean ± SD (n=3).

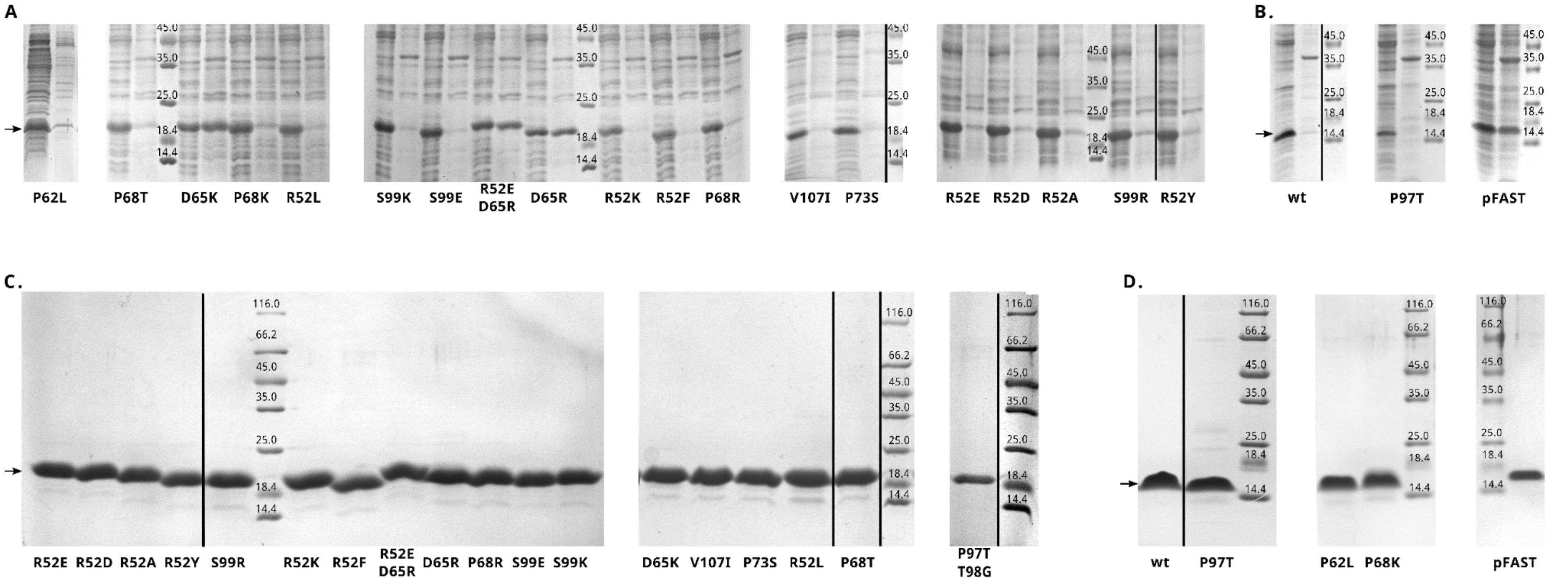

Supplementary Figure 25. Bacterially expressed and purified mutant variants of FAST used for fluorogenic properties tests. **A, B -** Cellular accumulation of FAST mutants. For each mutant a pair of soluble and insoluble cellular protein samples is applied to Tris-Glycine-SDS-PAGE (**A**) or Tris-Tricine-SDS-PAGE **(B).** An equivalent to 25 µl of M9 medium is loaded into each line. **C, D -** Purified FAST mutants. An aliquot of 2.5 µg of each variant (except for P97T-T98G and pFAST) is applied on Tris-Glycine-SDS-PAGE (**C**) or Tris-Tricine-SDS-PAGE (**D**). The protein bands corresponding to the FAST variants are indicated with an arrow. Black boundaries are placed between the lines if they are not adjacent in the same gel. The full-sized source gel images are presented in the Supplementary figure S29 for the cellular accumulation of all FAST mutants and in the Supplementary figure S30 for all the purified proteins. Protein molecular weight marker: 116.0, 66.2, 45.0, 35.0, 25.0, 18.4, 14.4 kDa Molecular weight of the wild type of FAST (wt) is 14.7kDa.

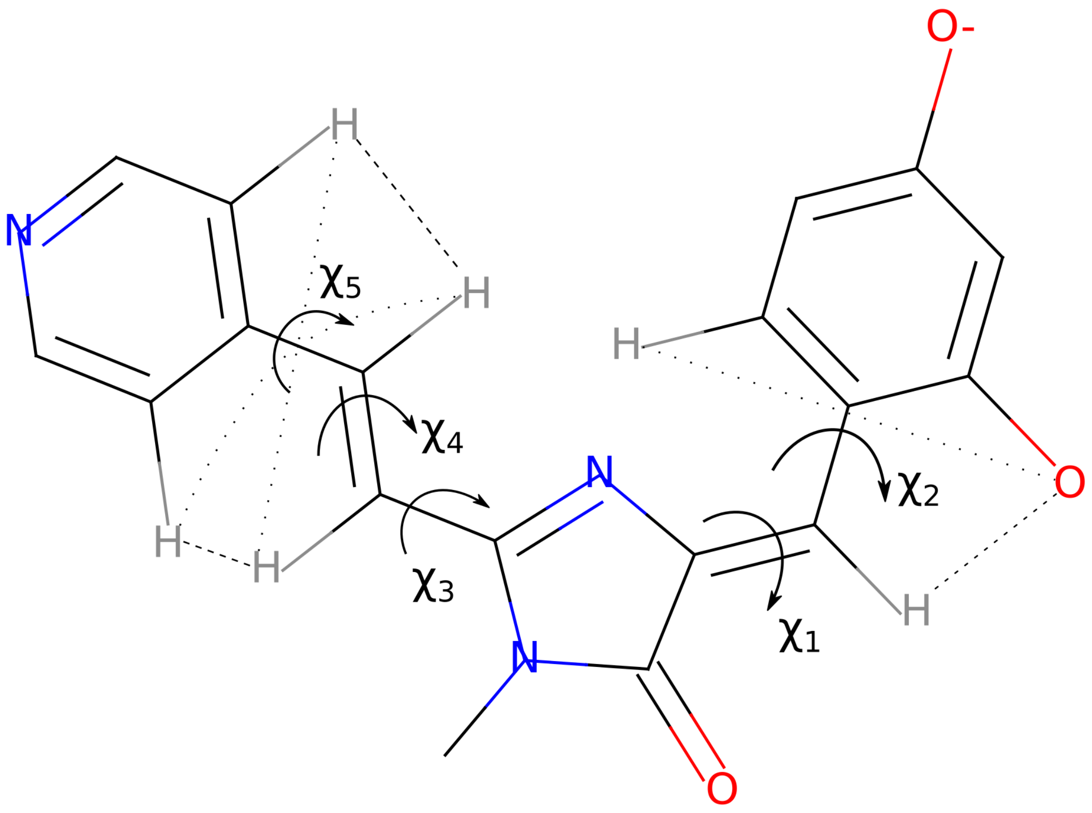

Supplementary Figure 26. The chemical structure of **N871b**. Torsion angles χ_1 - χ_5 and excluded nonbonded interactions are depicted. Nonbonded interactions that shift energy minimum from planar conformation are shown by dashed (this conformation) or dotted (after rotation of χ_2 or χ_5 by 180°) lines. Hydrogen atoms not included in excluded nonbonded list are not shown for clarity.

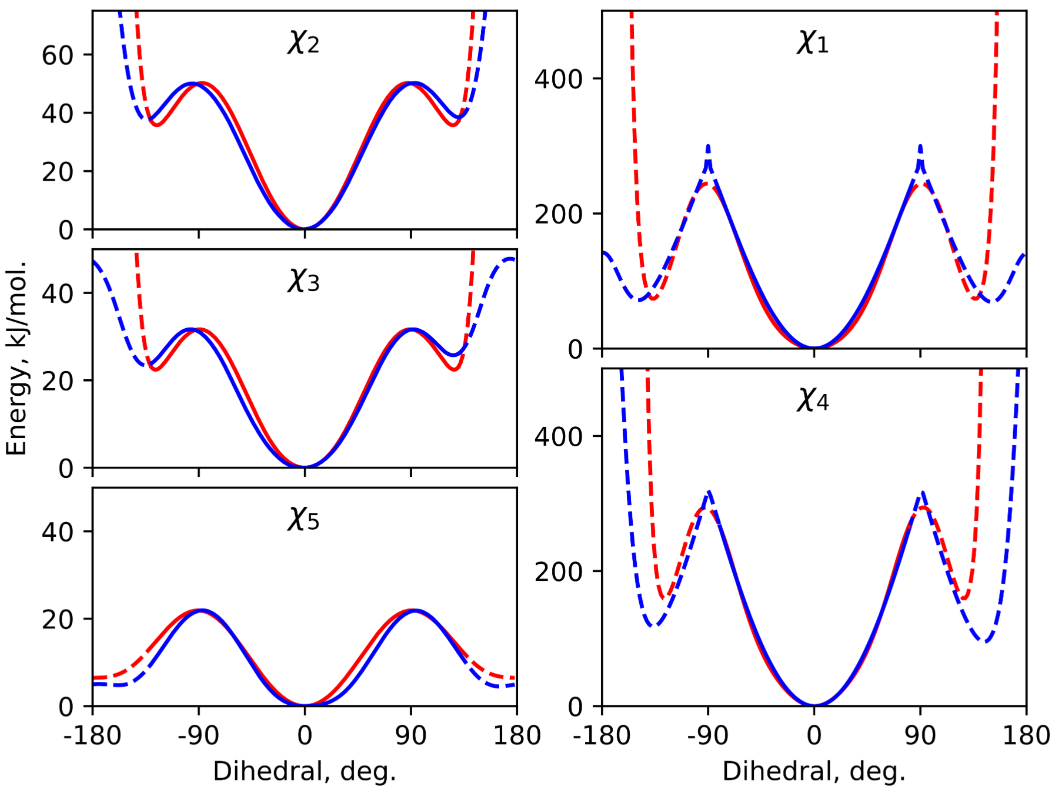

Supplementary Figure 27. The Gromacs (red) and GAMESS (blue) energies of **N871b** plotted versus dihedral angles. Energy profiles were calculated independently for dihedrals χ_1 – χ_5 starting from energy minimized planar conformation of **N871b** by rotating corresponding dihedral within range -180° – 180° with step of 2°. Dashed lines indicate regions where energy disturbed by nonbonded interactions. These regions were not taken into account during development of dihedral parameters for MD simulation of **N871b**.

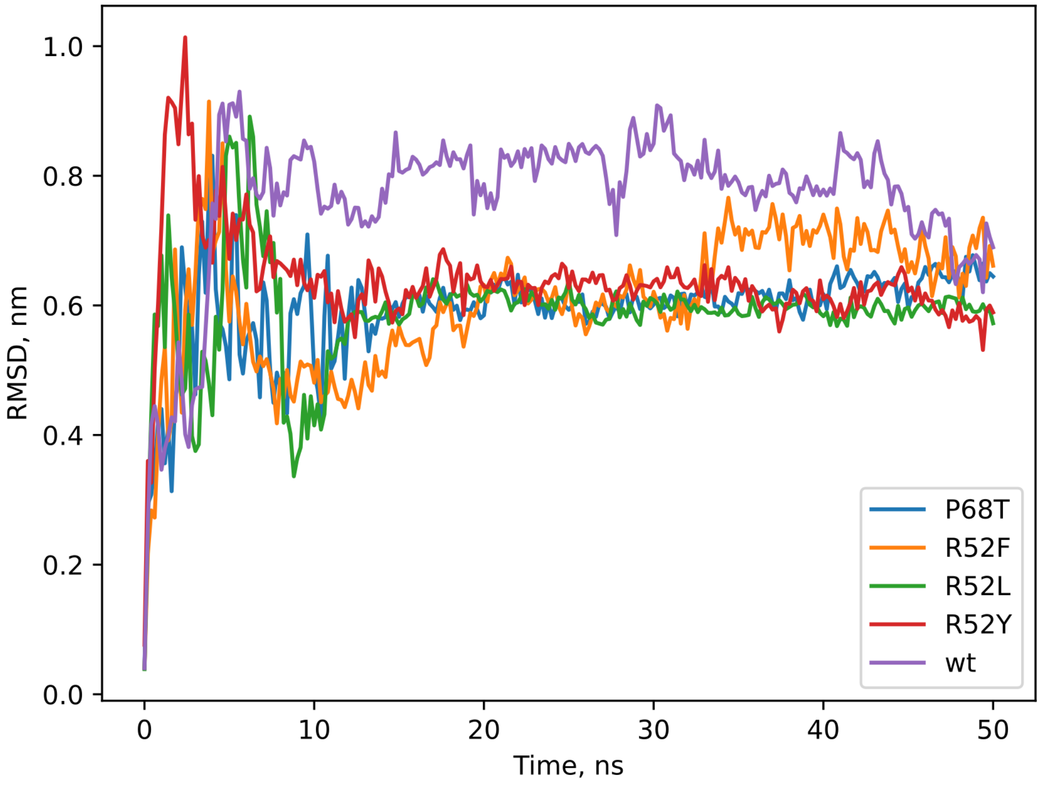

Supplementary Figure 28. RMSD of all atoms of protein-**N871b** complexes from starting (energy minimized) structure during equilibration stage of MD simulation. Result of MD simulation of FAST (model 1 in PDB ID 7AVA, wt) and its 4 mutants are shown.

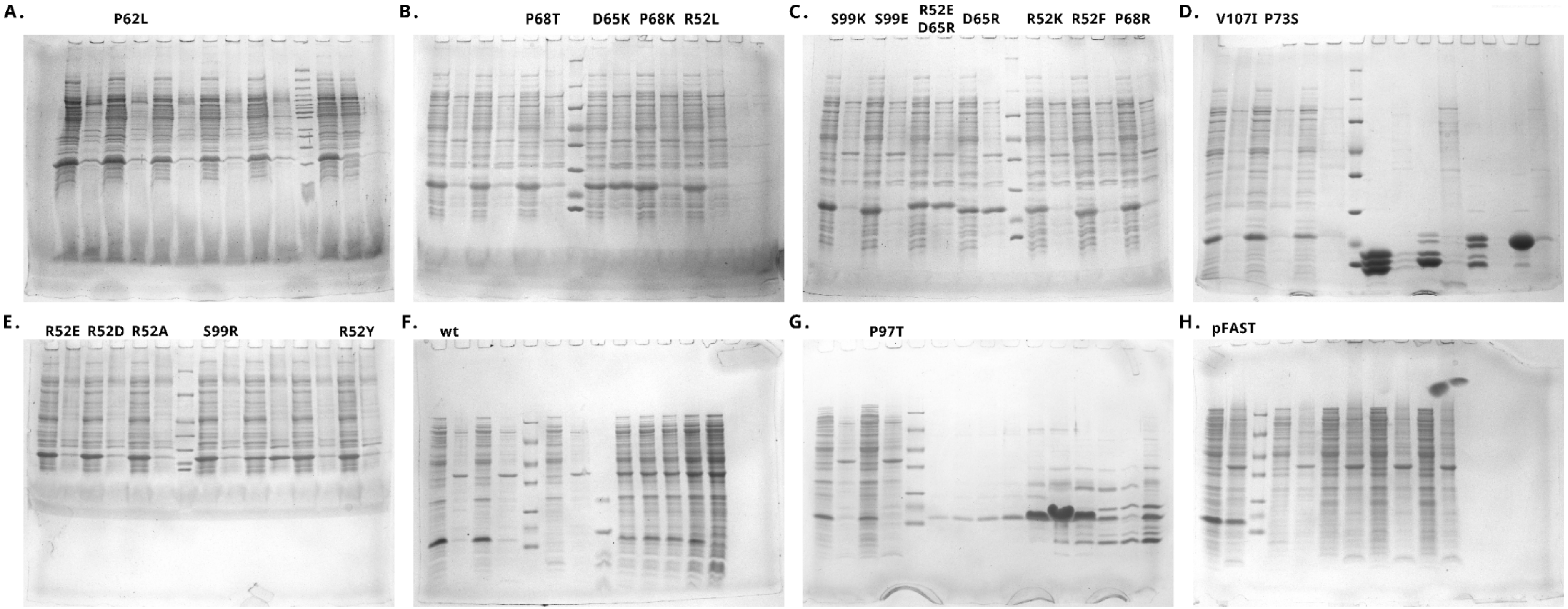

Supplementary Figure 29. The full-sized gels for cellular accumulation of FAST mutants shown in Supplementary Figure 25 (A, B). Appropriate lines are signed above the gel identically as they are in Supplementary Figure 25 (A, B). For each mutant a pair of soluble and insoluble cellular protein samples equivalent to 25 µl of M9 is applied to Tris-Glycine-SDS-PAGE (**A-E**) or Tris-Tricine-SDS-PAGE **(F-H).** Protein molecular weight markers - (**A):** 200, 150, 120, 100, 85, 70, 60, 50, 40, 30, 25, 20, 15, 10 kDa; **(B-H):** 116.0, 66.2, 45.0, 35.0, 25.0, 18.4, 14.4 kDa. Molecular weight of the wild type of FAST (wt) is 14.7kDa. The raw gels were combined into one figure and inscriptions were made using Inkscape 0.92 (https://www.inkscape.org).

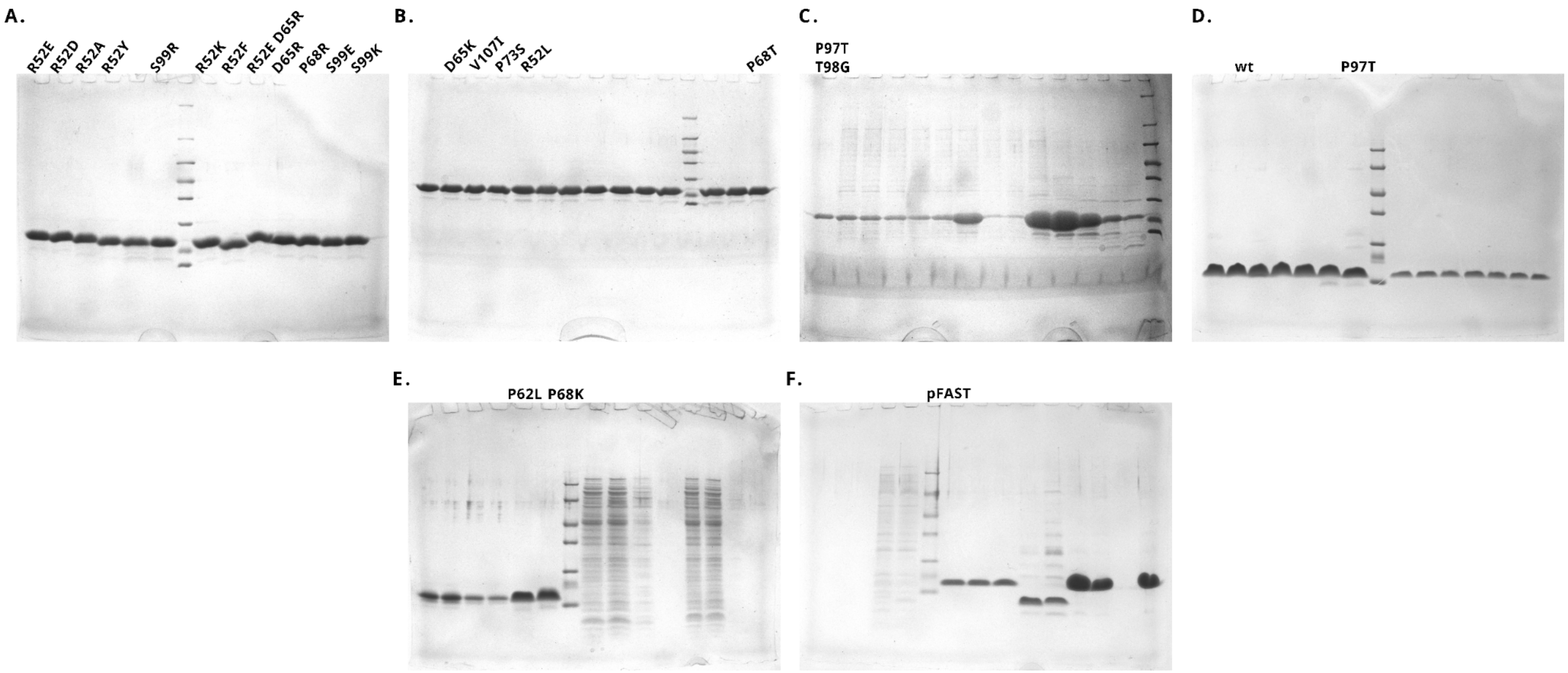

Supplementary Figure 30. The full-sized gels for purified FAST mutants shown in Supplementary Figure 25 (C, D). Appropriate lines are signed above the gel identically as they are in Supplementary Figure 25 (C, D). An aliquot of 2.5 µg of each variant (except for P97T-T98G and pFAST) is applied on Tris-Glycine-SDS-PAGE (**A-C**) or Tris-Tricine-SDS-PAGE (**D-F**). Protein molecular weight markers - 116.0, 66.2, 45.0, 35.0, 25.0, 18.4, 14.4 kDa. Molecular weight of the wild type of FAST (wt) is 14.7kDa. The raw gels were combined into one figure and inscriptions were made using Inkscape 0.92 (https://www.inkscape.org).

Supplementary Table 1. Amino acid sequences of the synthesized mutants. Amino acids which are extra to the FAST protein sequence are shown in brackets.

mutant	sequence[a]
**FAST WT**	(M)EHVAFGSEDIENTLAKMDDGQLDGLAFGAIQLDGDGNILQYNAAEGDITGRDPKQVIGKNFFKDVAPGTDSP<br>EFYGKFKEGVASGNLNTMFEWMIPTSRGPTKVKVHMKKALSGDSYWVFVKRV(KLAAALEHHHHHH)[b]
	(M)EHVAFGSEDIENTLAKMDDGQLDGLAFGAIQLDGDGNILQYNAAEGDITGRDPKQVIGKNFFKDVAPGTDSP<br>EFYGKFKEGVASGNLNTMFEWMIPTSRGPTKVKVHMKKALSGDSYWVFVKRV(GGGHHHHHH)[c]
**pFAST**	(M)EHVAFGSEDIENTLA**N**MDD**E**QLD**R**LAFG**V**IQLDGDGNIL**L**YNAAEGDITGRDPKQVIGKNFFKDVAPGTD**T**P<br>EFYGKFKEG**A**ASGNLNTMFEW**T**IPTSRGPTKVKVH**L**KKALSGD**R**YWVFVKRV(GGGHHHHHH)
**D65K**	(M)EHVAFGSEDIENTLAKMDDGQLDGLAFGAIQLDGDGNILQYNAAEGDITGRDPKQVIGKNFFK**K**VAPGTDSP<br>EFYGKFKEGVASGNLNTMFEWMIPTSRGPTKVKVHMKKALSGDSYWVFVKRV(GGGHHHHHH)
**D65R**	(M)EHVAFGSEDIENTLAKMDDGQLDGLAFGAIQLDGDGNILQYNAAEGDITGRDPKQVIGKNFFK**R**VAPGTDSP<br>EFYGKFKEGVASGNLNTMFEWMIPTSRGPTKVKVHMKKALSGDSYWVFVKRV(GGGHHHHHH)
**F62L**	(M)EHVAFGSEDIENTLAKMDDGQLDGLAFGAIQLDGDGNILQYNAAEGDITGRDPKQVIGKN**L**FKDVAPGTDSP<br>EFYGKFKEGVASGNLNTMFEWMIPTSRGPTKVKVHMKKALSGDSYWVFVKRV(GGGHHHHHH)
**P68K**	(M)EHVAFGSEDIENTLAKMDDGQLDGLAFGAIQLDGDGNILQYNAAEGDITGRDPKQVIGKNFFKDVA**K**GTDSP<br>EFYGKFKEGVASGNLNTMFEWMIPTSRGPTKVKVHMKKALSGDSYWVFVKRV(GGGHHHHHH)
**P68R**	(M)EHVAFGSEDIENTLAKMDDGQLDGLAFGAIQLDGDGNILQYNAAEGDITGRDPKQVIGKNFFKDVA**R**GTDSP<br>EFYGKFKEGVASGNLNTMFEWMIPTSRGPTKVKVHMKKALSGDSYWVFVKRV(GGGHHHHHH)
**P68T**	(M)EHVAFGSEDIENTLAKMDDGQLDGLAFGAIQLDGDGNILQYNAAEGDITGRDPKQVIGKNFFKDVA**T**GTDSP<br>EFYGKFKEGVASGNLNTMFEWMIPTSRGPTKVKVHMKKALSGDSYWVFVKRV(GGGHHHHHH)
**P73S**	(M)EHVAFGSEDIENTLAKMDDGQLDGLAFGAIQLDGDGNILQYNAAEGDITGRDPKQVIGKNFFKDVAPGTDS**S**<br>EFYGKFKEGVASGNLNTMFEWMIPTSRGPTKVKVHMKKALSGDSYWVFVKRV(GGGHHHHHH)

**P97T**	(M)EHVAFGSEDIENTLAKMDDGQLDGLAFGAIQLDGDGNILQYNAAEGDITGRDPKQVIGKNFFKDVAPGTDSP<br>EFYGKFKEGVASGNLNTMFEWMI**T**TSRGPTKVKVHMKKALSGDSYWVFVKRV(GGGHHHHHH)
**P97T/T98G**	(M)EHVAFGSEDIENTLAKMDDGQLDGLAFGAIQLDGDGNILQYNAAEGDITGRDPKQVIGKNFFKDVAPGTDSP<br>EFYGKFKEGVASGNLNTMFEWMI**TG**SRGPTKVKVHMKKALSGDSYWVFVKRV(GGGHHHHHH)
**R52A**	(M)EHVAFGSEDIENTLAKMDDGQLDGLAFGAIQLDGDGNILQYNAAEGDITG**A**DPKQVIGKNFFKDVAPGTDSP<br>EFYGKFKEGVASGNLNTMFEWMIPTSRGPTKVKVHMKKALSGDSYWVFVKRV(GGGHHHHHH)
**R52E**	(M)EHVAFGSEDIENTLAKMDDGQLDGLAFGAIQLDGDGNILQYNAAEGDITG**E**DPKQVIGKNFFKDVAPGTDSP<br>EFYGKFKEGVASGNLNTMFEWMIPTSRGPTKVKVHMKKALSGDSYWVFVKRV(GGGHHHHHH)
**R52E/D65R**	(M)EHVAFGSEDIENTLAKMDDGQLDGLAFGAIQLDGDGNILQYNAAEGDITG**E**DPKQVIGKNFFK**R**VAPGTDSP<br>EFYGKFKEGVASGNLNTMFEWMIPTSRGPTKVKVHMKKALSGDSYWVFVKRV(GGGHHHHHH)
**R52F**	(M)EHVAFGSEDIENTLAKMDDGQLDGLAFGAIQLDGDGNILQYNAAEGDITG**F**DPKQVIGKNFFKDVAPGTDSP<br>EFYGKFKEGVASGNLNTMFEWMIPTSRGPTKVKVHMKKALSGDSYWVFVKRV(GGGHHHHHH)
**R52K**	(M)EHVAFGSEDIENTLAKMDDGQLDGLAFGAIQLDGDGNILQYNAAEGDITG**K**DPKQVIGKNFFKDVAPGTDSP<br>EFYGKFKEGVASGNLNTMFEWMIPTSRGPTKVKVHMKKALSGDSYWVFVKRV(GGGHHHHHH)
**R52L**	(M)EHVAFGSEDIENTLAKMDDGQLDGLAFGAIQLDGDGNILQYNAAEGDITG**L**DPKQVIGKNFFKDVAPGTDSP<br>EFYGKFKEGVASGNLNTMFEWMIPTSRGPTKVKVHMKKALSGDSYWVFVKRV(GGGHHHHHH)
**R52Y**	(M)EHVAFGSEDIENTLAKMDDGQLDGLAFGAIQLDGDGNILQYNAAEGDITG**Y**DPKQVIGKNFFKDVAPGTDSP<br>EFYGKFKEGVASGNLNTMFEWMIPTSRGPTKVKVHMKKALSGDSYWVFVKRV(GGGHHHHHH)
**R52D**	(M)EHVAFGSEDIENTLAKMDDGQLDGLAFGAIQLDGDGNILQYNAAEGDITG**D**DPKQVIGKNFFKDVAPGTDSP<br>EFYGKFKEGVASGNLNTMFEWMIPTSRGPTKVKVHMKKALSGDSYWVFVKRV(GGGHHHHHH)
**S99E**	(M)EHVAFGSEDIENTLAKMDDGQLDGLAFGAIQLDGDGNILQYNAAEGDITGRDPKQVIGKNFFKDVAPGTDSP<br>EFYGKFKEGVASGNLNTMFEWMIPT**E**RGPTKVKVHMKKALSGDSYWVFVKRV(GGGHHHHHH)
**S99K**	(M)EHVAFGSEDIENTLAKMDDGQLDGLAFGAIQLDGDGNILQYNAAEGDITGRDPKQVIGKNFFKDVAPGTDSP<br>EFYGKFKEGVASGNLNTMFEWMIPT**K**RGPTKVKVHMKKALSGDSYWVFVKRV(GGGHHHHHH)
**S99R**	(M)EHVAFGSEDIENTLAKMDDGQLDGLAFGAIQLDGDGNILQYNAAEGDITGRDPKQVIGKNFFKDVAPGTDSP<br>EFYGKFKEGVASGNLNTMFEWMIPT**R**RGPTKVKVHMKKALSGDSYWVFVKRV(GGGHHHHHH)

**V107I**	(M)EHVAFGSEDIENTLAKMDDGQLDGLAFGAIQLDGDGNILQYNAAEGDITGRDPKQVIGKNFFKDVAPGTDSP<br>EFYGKFKEGVASGNLNTMFEWMIPTSRGPTKVK**I**HMKKALSGDSYWVFVKRV(GGGHHHHHH)

a – Aminoacids which are extra to the FAST protein sequence are shown in brackets. Mutated residues are shown in bold.

b – Aminoacid sequence of FAST WT used for NMR structural study.

c – Aminoacid sequence of FAST WT used for all the experiments.

Supplementary Table 2. Optical properties of complexes **N871b** with FAST mutants measured in PBS buffer.

Mutant	K_D, µM [a]	ε, M^{-1}·cm^{-1} [b]	Fluorescence quantum yield (FQY), % [c]	Brightness (=ε·FQY)	Absorbance maxima position, nm	Emission maxima position, nm
**FAST WT**	0.33±0.01	27000±410	26±1.4	7000±480	553	609
**D65K**	0.25±0.01	27500±410	26±2.1	7100±680	555	607
**D65R**	0.34±0.04	27000±410	24±1.2	6450±420	554	609
**F62L**	0.55±0.08	27500±410	19±0.9	5250±330	552	611
**P68K**	0.22±0.01	26500±400	18±0.7	4750±260	548	610
**P68R**	0.31±0.03	27000±410	23±0.4	6100±200	547	609
**P68T**	0.41±0.03	26500±400	17±1.5	4550±470	527	604
**P73S**	0.17±0.01	28000±420	20±1.2	5600±420	558	610
**P97T**	na [d]	na [d]	na [d]	na [d]	na [d]	na [d]
**P97T/T98G**	na [d]	na [d]	na [d]	na [d]	na [d]	na [d]
**R52A**	0.79±0.05	28500±430	27±1.0	7700±400	558	607
**R52E**	1.30±0.07	26000±390	26±0.2	6700±150	556	607
**R52E/D65R**	1.36±0.22	25000±380	26±1.5	6500±470	556	605
**R52F**	0.54±0.04	28000±420	23±0.4	6450±210	557	606
**R52K**	0.42±0.02	29500±440	24±1.2	7100±460	549	605
**R52L**	0.27±0.01	30000±450	29±1.7	8750±640	556	604
**R52Y**	0.24±0.02	29000±440	29±1.5	8350±560	558	605
**R52D**	na [d]	na [d]	na [d]	na [d]	na [d]	na [d]
**S99E**	0.21±0.01	28500±430	25±1.1	7050±420	556	608
**S99K**	0.27±0.01	28000±420	25±0.3	7050±190	556	609
**S99R**	0.24±0.01	28500±430	24±1.2	6800±440	555	608
**V107I**	0.38±0.05	26500±400	27±3.2	7150±950	555	607

a – represented as mean ± SD (n = 3);

b – represented as result of single measurement ± the precision of the measuring instruments (weighing and pipetting errors);

c – represented as mean ± SD (n = 9, except **R52E** and **R52E/D65R** where n = 3);

d – data was not obtained due to the poor fluorescence of the complex.

Supplementary Table 3. Optical properties of **N871b** in complexes with FAST mutants measured in various buffers.

Buffer	Mutant	K_D, μM [a]	ε, $M^{-1}\cdot cm^{-1}$ [b]	FQY, % [c]	Brightness
PBS buffer, pH 7.4, #cat E404-200TABS, Amresco	**FAST**	0.33±0.01	27000±410	26±1.4	7000±480
	**R52L**	0.27±0.01	30000±450	29±1.7	8750±640
	**R52Y**	0.24±0.02	29000±440	29±1.5	8350±560
pH 7.0, 20 mM NaPi, 20 mM NaCl	**FAST**	0.21±0.01	26000±390	29±1.1	7500±400
	**R52L**	0.19±0.02	28500±430	31±1.2	9000±480
	**R52Y**	0.17±0.03	27500±410	34±2.3	9400±780

a – represented as mean ± SD (n = 3);
b – represented as result of single measurement ± the precision of the measuring instruments (weighing and pipetting errors);
c – fluorescence quantum yield, represented as mean ± SD (n = 9).

Supplementary Table 4. NMR parameters of studied FAST/**N871b** complexes measured in PBS buffer.

	NMR parameters					
**mutant**	**E46 I[a]**	**E46 c.s.[b]**	**Y42 I**	**Y42 c.s.**	**W94 I**	**W94 c.s.**
**FAST WT**	0.20	14.92	0.06	12.69	0.74	12.05
**D65K**	0.20	14.91	0.04	12.77	0.77	12.01
**D65R**	0.21	14.91	0[c]		0.78	12.01
**F62L**	0.07	14.44	0.05	13.09	0.73	11.85
**P68K**	0.19	14.88	0.07	12.71	0.81	11.86
**P68R**	0.22	14.71	0.07	12.75	0.78	11.84
**P68T**	0.12	14.93	0		0.78	11.78
**P73S**	0.13	14.98	0.04	12.57	0.75	11.87
**P97T**	n.a.[d]					
**P97T/T98G**	0		0		0.36	11.57
**R52A**	0.06	15.04	0		0.77	12.07
**R52E**	0		0		0.67	12.18
**R52E/D65R**	0		0		0.58	12.15
**R52F**	0.26	15.23	0.38	12.76	0.74	12.17
**R52K**	0.11	14.86	0.12	13.15/12.68[e]	0.69	12.05
**R52L**	**0.26**	**15.03**	**0.45**	**12.63**	**0.76**	**12.14**
**R52Y**	**0.30**	**15.02**	**0.38**	**12.77**	**0.77**	**12.08**
**R52D**	0		0		0	
**S99E**	0.19	14.91	0.06	12.72	0.74	12.07
**S99K**	0.21	14.91	0.05	12.74	0.77	12.04
**S99R**	0.19	14.91	0.05	12.75	0.76	12.05
**V107I**	0		0		0.80	11.7

[a]I - integral of the corresponding signal in 1D NMR spectrum, relatively to ⅓ integral of the FAST methyl group signal at -0.66 ppm.

[b] Chemical shifts of Y42 Hη, E46 Hε2, and Hε1 W94 in ppm, measured at 25°C relative to the signal of TSP at 0.0 ppm.

[c]0 - the signal is not observed.

[d]data was not obtained, due to the low protein yields and poor fluorescence of the protein in complex with **N871b**.

[e]For R52K two states are observed with 1:1 intensity ratio. See Figure S3.

Supplementary Table 5. Mutations, found to enhance the activity of FAST, according to the literature.

target	name	mutations										
**HBR-3OM[12][a]**	iFAST	V107I[b]										
**HMBR[12]**	V122I	V122I										
**HMBR[13]**	gFAST	G21E	P68T	G77R								
**HMBR[13]**	gFAST1	F62L	P68S	T70K	Y76F	K80N						
**HMBR[13]**	gFAST2	P68T	T70K									
**HMBR[13]**	gFAST3	S8R	F62L	P68H	T70P	K80N						
**HMBR[13]**	gFAST4	P68T	F75L	E93D								
**HMBR[13]**	gFAST5	G35S	D36G	S72T	E93D	V107M						
**HMBR[13]**	gFAST6	P68T	T70R									
**HMBR[13]**	gFAST7	Q41R	E93D	V107M								
**HMBR[13]**	gFAST8	P68T	T70K	E93V	G115S							
**HBR-3,5DOM[13]**	rFAST	F28L	E46Q	R52A	E81V	S99N						
**HBR-3,5DOM[13]**	rFAST1	K17R	D19G	F28L	A30T	E46Q	K60R					
**HBR-3,5DOM[13]**	rFAST2	A30V	R52S	K60R	V83A	K111R	S117C	Y118F				
**HBR-3,5DOM[13]**	rFAST3	G21R	F28L	E46Q								
**HBR-3,5DOM[13]**	rFAST4	F28L	E46Q	S117R								
**HBR-3,5DOM[13]**	rfAST5	L33F	Q41H	E46Q	K111N							
**HBR-3,5DOM[13]**	rFAST6	R52A	K80M	S99I								
**HBR-3,5DOM[13]**	rFAST7	R52A	E81V	S99N								
**HBR-3,5DOM[13]**	rFAST8	D20H	F28I	E46Q								
**HBO-3M[10]**	AR68	D65V	E93D	M109L	S117R							
**HBO-3M[10]**	AR611	V83I	M109L									
**HBO-3M[10]**	AR71	M109L										
**HBO-3M[10]**	AR72	Q41L	M109L									
**HBO-3M[10]**	AR710	V83I	T103I									
**HBO-3M[10]**	AR712	K17R	A30V	E74K	E93V							
**HBO-3M[10]**	AR714	T50S	E93Q	M95I								
**HPAR-3OM[11]**	frFAST	F62L	D71V	P73S	E74G	V107I						
**HBP-3,5DM[10]**	AR53	Q41H	V83L	M95I								
**HBP-3,5DM[10]**	AR57	Q41K	S72T	V83A	M95I							
**HBP-3,5DM[10]**	AR512	K17N	Q41K	M95T								
**HBP-3,5DM[10]**	AR513	Q41L	S117I									
**HBP-3,5DM[10]**	AR521	V83E	S117R									
**HBP-3,5DM[10]**	AR62	E93G	M95V	M109L								
**HBP-3,5DM[10]**	AR71	D20N	E81Q	V83I	M109L							
**HBP-3,5DM[10]**	AR76	G25R	A84S									
**HBP-3,5DM[10]**	AR710	Q32K	Y76N									
**HBP-3,5DM[10]**	BR52	K17N	Q41K	S72T	M95T							
**HBP-3,5DM[10]**	BR511	K17N	G25E	S72T	V83A	M95T						
**HBP-3,5DM[10]**	BR519	Q41K	K80M	A84S	N89D	M95T	M109L					
**HBP-3,5DM[10]**	BR61	K17N	G21E	G25R	A30V	Q41L	S72T	V83A	M95T	S117R		
**HBP-3,5DM[10]**	BR610	Q41K	S72T	V83A	S117R							
**HBP-3,5DM[10]**	BR615	N13S	Q41K	S72T	V83A	M95T						

**HBP-3,5DM[10]**	BR619	K17I	G25R	Q41K	A44T	K60R	V38A	N89D	E93K	M95T	M109L	S117R
**HBP-3,5DM[10]**	BR73	K17N	Q41K	D65E	S72T	V83A	K106M	M109L				
**HBT-3,5DM[10]**	BR611	A16D	Q41K	Y76H	V83A	M95T	M109L	S117R				
**HBT-3,5DM[10]**	tFAST	G25R	Q41K	S72T	A84S	M95A	M109L	S117R				
**HBT-3,5DM[10]**	BR617	A27V	Q41K	S72T	A84S	M95A	M109L	S117R				
**HBT-3,5DM[10]**	BR624	K17N	G21E	G25R	A30V	Q41L	S72T	V83A	M95T	M109L		
**HBT-3,5DM[10]**	BR72	Q41K	K80R	V83A	M95T	M109L						
**HBT-3,5DM[10]**	BR74	Q41K	M95T	S117I								
**HBO-3,5DOM[10]**	BR67	G7D	Q41K	S72T	A84S	M95I	M109L	S117R	R124L			
**HBO-3,5DOM[10]**	BR73	K17N	Q41K	A84S	M95T	M109L	S117R					
**HBO-3,5DOM[10]**	BR77	Q41K	D48G	S72T	V83A	M95T	M109L	S117R				
**HBO-3,5DOM[10]**	BR722	G21R	G25R	Q41K	Q60E	M95T	M109L	A112G				
**HMBR[14]**	mutant1	F62L	P68C	D71R	P73S							
**HMBR[14]**	mutant2	D19N	F62L	P68C	D71R	P73S	M95T	S99K				
**HMBR[14]**	mutant3	F62L	P68E	D71R	P73S	M95T	S99K					

[a]Reference number of the paper, citing the provided FAST mutant is given in parentheses.

[b]Residues that take part in direct interactions with the ligand are colored red. Residues that are located in two unstable N-terminal helices of FAST or at the interface between the N-terminus and the major protein core are colored blue.
