# Supplementary material for: Structure-based rational design of an enhanced fluorogen-activating protein for fluorogens based on GFP chromophore
Source: Commun Biol. 2022 Jul 15;5:706. doi: 10.1038/s42003-022-03662-9 (PMC9287381; doi:10.1038/s42003-022-03662-9)
Supplement: Supplementary file 3 — Description of Additional Supplementary Files [file 42003_2022_3662_MOESM3_ESM.pdf]

Description of Additional Supplementary Files

File name: Supplementary Data 1
Description: Raw data behind figures 3 and 4.
