# Supplementary material for: Structure-based rational design of an enhanced fluorogen-activating protein for fluorogens based on GFP chromophore
Source: Commun Biol. 2022 Jul 15;5:706. doi: 10.1038/s42003-022-03662-9 (PMC9287381; doi:10.1038/s42003-022-03662-9)
Supplement: Supplementary file 5 — Reporting Summary [file 42003_2022_3662_MOESM5_ESM.pdf]

nature research

Corresponding author(s): Konstantin S. Mineev

Last updated by author(s): Jun 7, 2022

Reporting Summary

Nature Research wishes to improve the reproducibility of the work that we publish. This form provides structure for consistency and transparency in reporting. For further information on Nature Research policies, see our Editorial Policies and the Editorial Policy Checklist.

Statistics

For all statistical analyses, confirm that the following items are present in the figure legend, table legend, main text, or Methods section.

n/a	Confirmed	
☐	☒	The exact sample size (n) for each experimental group/condition, given as a discrete number and unit of measurement
☐	☒	A statement on whether measurements were taken from distinct samples or whether the same sample was measured repeatedly
☐	☒	The statistical test(s) used AND whether they are one- or two-sided<br>*Only common tests should be described solely by name; describe more complex techniques in the Methods section.*
☒	☐	A description of all covariates tested
☒	☐	A description of any assumptions or corrections, such as tests of normality and adjustment for multiple comparisons
☐	☒	A full description of the statistical parameters including central tendency (e.g. means) or other basic estimates (e.g. regression coefficient) AND variation (e.g. standard deviation) or associated estimates of uncertainty (e.g. confidence intervals)
☐	☒	For null hypothesis testing, the test statistic (e.g. F, t, r) with confidence intervals, effect sizes, degrees of freedom and P value noted<br>*Give P values as exact values whenever suitable.*
☒	☐	For Bayesian analysis, information on the choice of priors and Markov chain Monte Carlo settings
☒	☐	For hierarchical and complex designs, identification of the appropriate level for tests and full reporting of outcomes
☒	☐	Estimates of effect sizes (e.g. Cohen's d, Pearson's r), indicating how they were calculated

Our web collection on statistics for biologists contains articles on many of the points above.

Software and code

Policy information about availability of computer code

Data collection	Bruker Topspin 3.5, Varian UV Scan Application 3.0, Cary Eclipse Scan Applications 1.2, Tecan i-control 3.7.3.0, Micro-Manager 1.4.23, FijiImageJ 1.52
Data analysis	Wolfram Mathematica 5.0, Pymol, Gromacs, Origin 8.6, Microsoft Office 2013, Amber Tools 2020, GAMESS, JMol, PR.Panta Analysis v1.2, Tensor 2, CARA

For manuscripts utilizing custom algorithms or software that are central to the research but not yet described in published literature, software must be made available to editors and reviewers. We strongly encourage code deposition in a community repository (e.g. GitHub). See the Nature Research guidelines for submitting code & software for further information.

Data

Policy information about availability of data

All manuscripts must include a data availability statement. This statement should provide the following information, where applicable:
- Accession codes, unique identifiers, or web links for publicly available datasets
- A list of figures that have associated raw data
- A description of any restrictions on data availability

All the data are available on request to the corresponding authors. Unedited SDS-PAGE gels are provided as supplementary figures 29 and 30. Raw data that was used for the charts in Figures 3 and 4 are provided as supplementary data file 1.

Field-specific reporting

Please select the one below that is the best fit for your research. If you are not sure, read the appropriate sections before making your selection.

☒ Life sciences ☐ Behavioural & social sciences ☐ Ecological, evolutionary & environmental sciences

For a reference copy of the document with all sections, see nature.com/documents/nr-reporting-summary-flat.pdf

Life sciences study design

All studies must disclose on these points even when the disclosure is negative.

Sample size	Sample size was taken n=9 for the in vitro analysis of the mutant brightness and n=29 for the analysis of in-cell brightness. The sample size was sufficient to get the estimate of statistical reliability of the observed effects
Data exclusions	No data points were excluded
Replication	Brightnesses of the parent protein and most prospective mutants were measured at least two times on the independently synthesized protein samples and in two different buffers at pH 7.0 and 7.5. All the replication attempts were successful
Randomization	not applicable
Blinding	Blinding is not relevant for the current study, since there were no experiments with the results that can depend on the experimentator

Reporting for specific materials, systems and methods

We require information from authors about some types of materials, experimental systems and methods used in many studies. Here, indicate whether each material, system or method listed is relevant to your study. If you are not sure if a list item applies to your research, read the appropriate section before selecting a response.

Materials & experimental systems

n/a	Involved in the study	
☒	☐	Antibodies
☐	☒	Eukaryotic cell lines
☒	☐	Palaeontology and archaeology
☒	☐	Animals and other organisms
☒	☐	Human research participants
☒	☐	Clinical data
☒	☐	Dual use research of concern

Methods

n/a	Involved in the study	
☒	☐	ChIP-seq
☒	☐	Flow cytometry
☒	☐	MRI-based neuroimaging

Eukaryotic cell lines

Policy information about cell lines

Cell line source(s)	HeLa Kyoto cells were obtained from previously established stock of our laboratory
Authentication	HeLa Kyoto cells were authentificated morphologically
Mycoplasma contamination	HeLa Kyoto cells were tested negatively for mycoplasma contamination via commertial PCR kit
Commonly misidentified lines (See ICLAC register)	None
